# Picornavirus infection induces temporal release of multiple extracellular vesicle subsets that differ in molecular composition and infectious potential

**DOI:** 10.1371/journal.ppat.1007594

**Published:** 2019-02-19

**Authors:** Susanne G. van der Grein, Kyra A. Y. Defourny, Huib H. Rabouw, Chenna R. Galiveti, Martijn A. Langereis, Marca H. M. Wauben, Ger J. A. Arkesteijn, Frank J. M. van Kuppeveld, Esther N. M. Nolte-‘t Hoen

**Affiliations:** 1 Department of Biochemistry & Cell Biology, Faculty of Veterinary Medicine, Utrecht University, Utrecht, The Netherlands; 2 Department of Infectious Diseases & Immunity, Division of Virology, Faculty of Veterinary Medicine, Utrecht University, Utrecht, The Netherlands; 3 Department of Infectious Diseases & Immunity, Division of Immunology, Faculty of Veterinary Medicine, Utrecht University, Utrecht, The Netherlands; University of Pittsburgh, UNITED STATES

## Abstract

Several naked virus species, including members of the Picornaviridae family, have recently been described to escape their host cells and spread infection via enclosure in extracellular vesicles (EV). EV are 50–300 nm sized lipid membrane-enclosed particles produced by all cells that are broadly recognized for playing regulatory roles in numerous (patho)physiological processes, including viral infection. Both pro- and antiviral functions have been ascribed to EV released by virus-infected cells. It is currently not known whether this reported functional diversity is a result of the release of multiple virus-containing and non-virus containing EV subpopulations that differ in composition and function. Using encephalomyocarditis virus infection (EMCV, Picornaviridae family), we here provide evidence that EV populations released by infected cells are highly heterogeneous. Virus was contained in two distinct EV populations that differed in physical characteristics, such as sedimentation properties, and in enrichment for proteins indicative of different EV biogenesis pathways, such as the plasma membrane resident proteins Flotillin-1 and CD9, and the autophagy regulatory protein LC3. Additional levels of EV heterogeneity were identified using high-resolution flow cytometric analysis of single EV. Importantly, we demonstrate that EV subsets released during EMCV infection varied largely in potency of transferring virus infection and in their kinetics of release from infected cells. These data support the notion that heterogeneous EV populations released by virus-infected cells can exert diverse functions at distinct time points during infection. Unraveling the compositional, temporal and functional heterogeneity of these EV populations using single EV analysis technologies, as employed in this study, is vital to understanding the role of EV in virus dissemination and antiviral host responses.

## Author summary

Picornaviruses constitute a family of viruses that cause many human and animal diseases. These ‘naked’ viruses are known to spread by causing infected cell to burst open. However, challenging recent data indicate that virions also escape intact cells enclosed in small lipid membrane-enclosed particles, called ‘extracellular vesicles’ (EV). Inside these cell-derived EV, virions can stay under the radar of the host’s immune system, but there is also evidence that EV can facilitate anti-viral immune responses. We therefore hypothesized that different types of EV are released by naked virus-infected cells. Testing this hypothesis required advanced methods with which the very small EV (50–300 nm) can be individually characterized. We investigated the pool of EV released by cells infected with encephalomyocarditis virus (EMCV) using an in-house developed method for analysis and isolation of individual EV. We demonstrate that EMCV infection induces the release of several EV subpopulations that differ in the molecules they carry, the timing of their release from the infected cell, and in potency to transfer virus infection. Our discovery that EV released by naked virus-infected cells vary in composition and function is a critical step in further unraveling the role of EV in virus spreading and virus-host interactions.

## Introduction

Recent discoveries indicate that several naked virus species can escape from intact cells via a non-lytic release mechanism involving enclosure in membranous structures that resemble extracellular vesicles (EV) [1–4]. EV are submicron-sized (50–300 nm) lipid bilayer-enclosed particles containing proteins and RNA, and are increasingly recognized as an important means of intercellular communication employed by all cells (reviewed in [5]). EV can play a role in maintaining homeostasis as well as in various pathologies via the delivery of cargo molecules that trigger a response in distant or neighboring recipient cells (reviewed in [6]). Enclosure of naked virus particles in EV has been predominantly observed for viruses belonging to the Picornaviridae family, a group of small RNA viruses implicated in many human and veterinary diseases. Examples include members of the genus *Enterovirus*, such as poliovirus, coxsackievirus B3 (CVB3), and enterovirus 71 (EV71), as well as hepatitis A virus (HAV, genus *Hepatovirus*) [1,2,4,7]. Furthermore, the phylogenetically distinct hepatitis E virus (HEV, family Hepeviridae), which like HAV is a hepatotropic virus, has been shown to leave cells in an EV-enclosed form [8]. EV-enclosed naked viruses have also been observed *in vivo*, for example in serum of HEV and HAV-infected individuals [4,9,10]. Enclosure of virus particles in EV can benefit virus infections in several ways. First, the EV can shield the virus from immune recognition [4,7,11,12]. In addition, virus exit from host cells without inducing cell rupture limits tissue damage and consequent alarming of the immune system. Moreover, there is strong evidence that virus-containing EV can transfer the infection to new host cells [1,7]. The co-transfer of host molecules may influence the uptake of virus-containing EV, as has been described for phosphatidylserine (PS) lipids that contribute to cellular entry of EV-enclosed poliovirus and HAV [1,13,14]. Furthermore, the enclosure of multiple virions per EV could benefit infection by promoting cooperativity between genetic quasi-species [1]. On the contrary, EV-mediated release of virus or host products from infected cells can also trigger the antiviral immune response in EV-targeted cells that are non-susceptible or non-permissive to infection with the naked virus [13]. The reported pro- and antiviral effects of virus-induced EV could be explained by experimental variation between studies, e.g. with regard to virus strains, producer and recipient cell types, and EV isolation methods [15]. Alternatively, multiple EV populations with variable composition and function could be released by infected cells.

Heterogeneity is an intrinsic feature of EV populations released by cells under various culture conditions. This heterogeneity is increased even further by external stimuli imposed on the producer cells that alter the cell’s activation or differentiation state. Factors known to influence the quantity and quality of released EV include receptor-mediated stimulation, tumorigenic transformation, or environmental conditions such as hypoxia or nutrient-starvation [16–19]. Furthermore, EV that differ in size and/or molecular composition can be formed via distinct EV biogenesis routes involving either late endosomal compartments or the plasma membrane (reviewed in [5]). The formation routes of naked virus-containing EV are largely unknown and may be virus-type specific. Formation of EV-enclosed enteroviruses has been suggested to involve secretory autophagy, whereby virion-containing autophagosomes fuse with the plasma membrane to release their contents [1,20]. HAV- and HEV-containing EV, on the contrary, are proposedly formed via inward budding into endosomal compartments, and released upon fusion of these multivesicular bodies (MVBs) with the plasma membrane [4,8,11,21,22]. These data suggest that different virus types are released from cells enclosed in EV that originate from different biogenesis pathways. However, potential heterogeneity in molecular contents and function of EV within populations released by naked virus-infected cells has not yet been addressed. Furthermore, the virus-containing EV are likely produced against a background of constitutively released EV that do not enclose virus. To unravel the role of EV in naked virus infections, it is therefore crucial to gain in-depth knowledge on the complexity of EV populations released by infected cells.

Detection and characterization of naked virus-containing EV has until now mostly been performed by analyzing the presence of EV-related host proteins and viral components in bulk isolates of EV or by low-throughput electron microscopic analysis. These methodologies do not allow accurate assessment of EV population heterogeneity, because this requires high-throughput techniques that detect and analyze EV at the single particle level. Here, we employed an in-house developed high-resolution flow cytometric approach for qualitative and quantitative analysis of single EV to study EV release from naked virus-infected cells [23,24]. We used this technique in combination with infectivity assays and analysis of EV-associated proteins to investigate whether virions were contained in different EV types, and how the release of different EV subpopulations changed over the course of infection. In addition, we used high-resolution flow cytometric sorting of single EV to study how efficiently distinct EV subpopulations could transfer virus infection. To study structural and functional heterogeneity in EV populations released by naked virus-infected cells we used encephalomyocarditis virus (EMCV) of the *Cardiovirus* genus as a model picornavirus with a rapid lytic life cycle. EMCV is a 30 nm sized virus that can cause a variety of symptoms and diseases in a broad range of mammals [25].

Our data demonstrate that EMCV-infected cells release two distinct EV populations containing infectious virus, which differed in sedimentation properties and protein composition. Importantly, we obtained evidence for functional diversity in virus-induced EV by showing that EV subsets differed in potency of transferring infection to new host cells. Finally, we show that temporal release of the different subpopulations of EV is tightly regulated over the course of infection. These data illustrate that there are multiple levels of heterogeneity in EV released by naked virus-infected cells.

## Results

### EMCV is released via a non-lytic route in the early stage of infection

**Fig 1:**
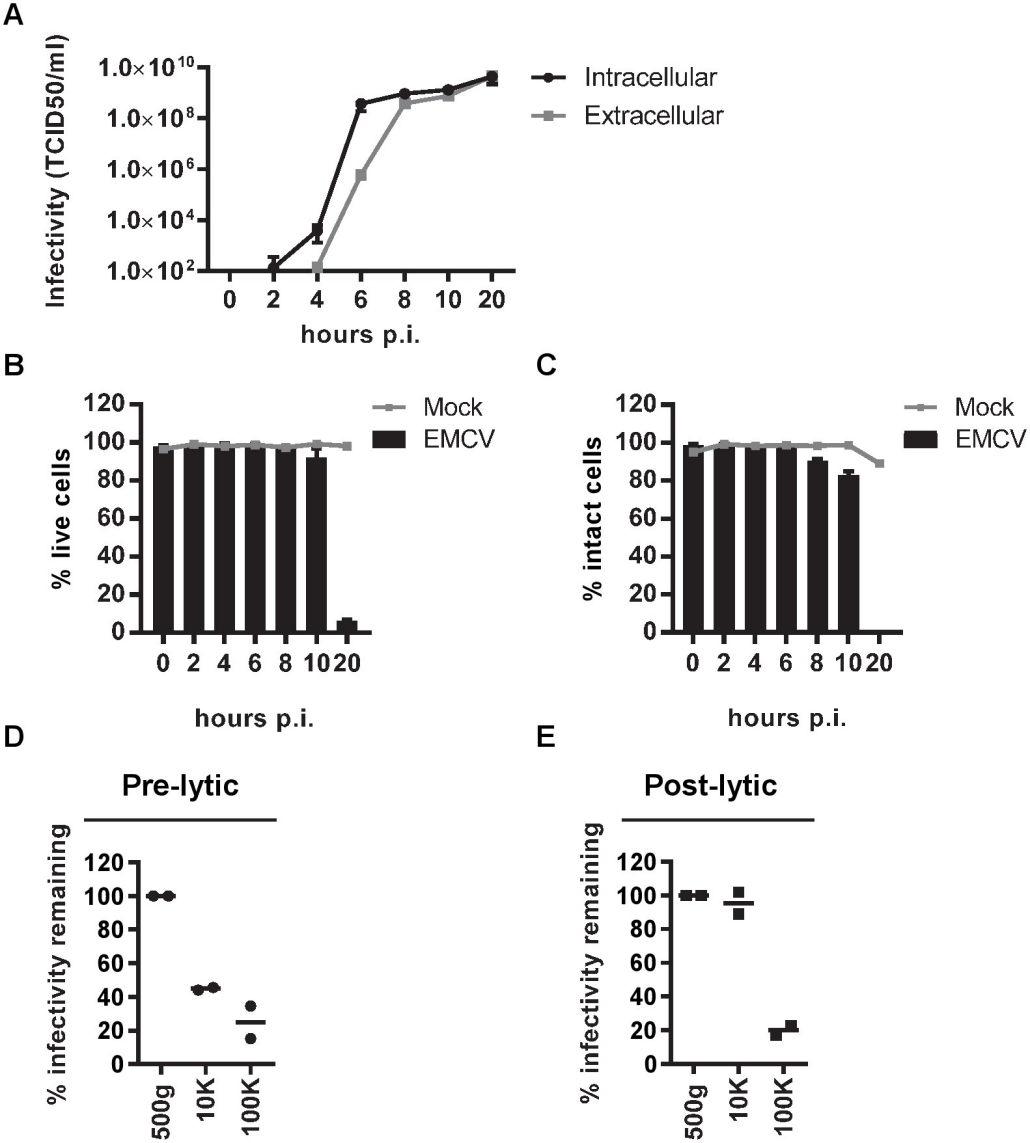
EMCV-infected cells release infectious particles that sediment at 10,000xg or 100,000xg in the pre-lytic phase of infection. (A-C): Mock and EMCV-infected HeLa cells (MOI = 10) and corresponding culture supernatants were harvested at various time points after infection. Line graphs display the increase over time of intracellular virus production and extracellular virus release determined by end-point dilution (A). Bars depict cell viability measured by fluorescent staining of dead cells (B) and cell integrity determined by measuring leakage of an intracellular enzyme into the extracellular space (C) at indicated time points after infection compared to mock-infected cells (grey lines). Mean values ± s.d. for N = 3 experiments are presented for A-C. (D) Supernatant from cells infected with EMCV for 8 hours was cleared from cells and debris by centrifugation at 500x*g*. Hereafter, infectious particles were pelleted by sequential high-speed ultracentrifugation steps at 10,000x*g* (10K) and 100,000x*g* (100K). The percentage of infectivity remaining in the supernatant after each centrifugation step was assessed by end-point dilution. (E) Naked EMCV virions harvested during the lytic phase of infection (20 hours p.i.) and depleted from membrane vesicles by treatment with 0.1% triton, were subjected to sequential centrifugation steps at 500x*g*, 10K, and 100K. The supernatant after each centrifugation step was assessed for remaining infectivity by end-point dilution. Infectivity in the supernatant after the 500x*g* centrifugation step was set to 100%. Data and mean values of two independent experiments are presented for both (D) and (E).

We first investigated whether non-lytic virus release occurred after infection with EMCV. Hereto, HeLa cells were infected with a high multiplicity of infection (MOI = 10) and virus production and release were monitored over the course of infection. The earliest time point at which detectable levels of intracellular virus were observed was 4 hours post-infection (p.i.). From 6 hours p.i. onwards, virus could be detected outside cells in the culture supernatant (Fig 1A). In parallel we monitored cell viability and plasma membrane integrity over the course of infection by using flow cytometric analysis of cells stained with a fixable viability dye that labels cells with compromised plasma membranes (Fig 1B, S1 Fig). In addition, we performed assays measuring leakage of the intracellular enzyme LDH into the extracellular space (Fig 1C). While at 20 hours p.i. complete cell disintegration had occurred, up to 10 hours p.i. minimal signs of cell death or loss of plasma membrane integrity were observed. In subsequent experiments, non-lytic virus release was therefore studied during the first 8 hours of EMCV infection.

### During the pre-lytic phase of infection, cells release infectious virus in multiple types of EV

We next questioned whether infectious virus released during pre-lytic EMCV infection was contained in EV. In the field of EV research, a centrifugal force of ~100,000x*g* is often applied to collect EV from body fluids and cell culture supernatant. However, recent research has demonstrated that viable cells also release functionally active EV of larger size (up to 1 μm) that sediment at ~10,000x*g* [26–28]. In previous studies on EV-enclosed naked viruses, these low-*g*-force sedimenting large EV were often either co-isolated with smaller EV or were discarded [15]. To assess whether EMCV was contained in large or small EV, culture supernatants of infected cells (8 hours p.i.) were subjected to consecutive centrifugation steps of 10,000x*g* (10K) and 100,000x*g* (100K). The percentage of infectivity remaining in the supernatant was determined after each of these steps. Centrifugation at 10K for 30 minutes depleted the supernatant of a large portion of the released infectivity whereas subsequent centrifugation at 100K for 1 hour further reduced the amount of infectious material (Fig 1D). With high-speed ultracentrifugation, also naked virus particles that overlap in size with small EV may sediment. We investigated this by applying the same centrifugal forces to virions derived from post-lytic culture supernatants, from which membranous structures were removed by detergent treatment. Naked EMCV virions sedimented at 100K, but not at 10K (Fig 1E). Hence, the infectivity of particles pelleted at 10K in the pre-lytic supernatant cannot be attributed to naked virus, while the 100K pellets of this material could contain both naked and EV-enclosed infectious virus.

**Fig 2:**
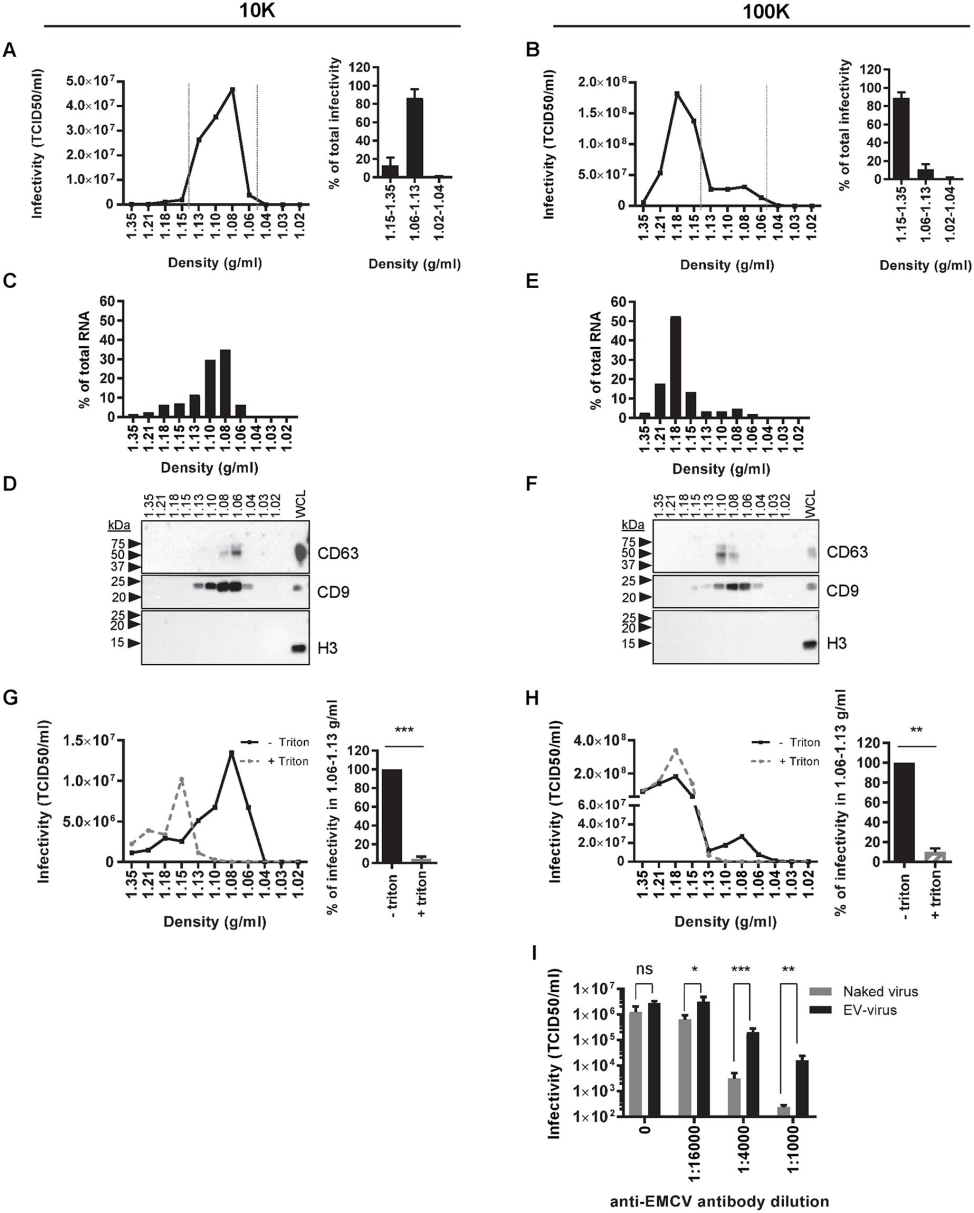
Virus particles released during pre-lytic EMCV infection are enclosed in two EV subpopulations. (A-F): 10K (A,C,D) and 100K (B,E,F) pelleted particles from the supernatant of cells infected for 8 hours with EMCV were separated by buoyant density gradient centrifugation. The level of infectivity in individual gradient fractions was assessed by end-point dilution (A,B left panels). Bar graphs (A, B right panels) indicate the relative distribution of infectivity in the 1.15–1.35 g/ml, 1.06–1.13 g/ml, and 1.02–1.04 g/ml density segments (N = 4 and N = 9 individual experiments for 10K and 100K fractions, respectively). (C, E) Indicated is the amount of viral genomic RNA, as assessed by RT-qPCR, in the individual fractions expressed as a percentage of total viral RNA in the whole gradient. (D, F) Density gradient fractions and whole cell lysates (WCL) were analyzed for the presence of CD63, CD9 and histon protein H3 by western blotting. (G-H) 10K (G) and 100K (H) centrifugation pellets from the supernatant of EMCV-infected cells 8 hrs p.i. were treated with 0.1% triton prior to separation on density gradients. The level of infectivity in individual gradient fractions was assessed by end-point dilution (left panels). Bar graphs (right panels) indicate the infectivity present in the 1.06–1.13 g/ml density segment in 10K and 100K samples after triton treatment and in control conditions (set to 100%). Indicated are mean values ± s.d. for N = 3 independent experiments (** p<0.005, *** p<0.0005). (I) The level of infectivity present in the 1.08 g/ml (EV-enclosed virus) and 1.21 g/ml (naked virus) 100K density fractions in the absence or presence of indicated dilutions of anti-EMCV capsid antibody was determined by end-point dilution. Presented are mean values ± s.d. for N = 3 experiments (ns = not significant, * p<0.05, ** p<0.005, *** p<0.0005).

To further characterize the infectious particles pelleted at 10K or 100K in pre-lytic culture supernatant, we employed isopycnic density gradient centrifugation to distinguish between lipid bilayer-surrounded EV and naked virions [4]. Using this method, we confirmed that the buoyant density of naked EMCV virions was considerably higher (1.18 g/ml) than the density of EV released by the non-infected HeLa cells (1.06–1.13 g/ml) (S2A and S2B Fig). To investigate whether infectious virus was present in EV released during the pre-lytic phase, we separated 10K and 100K pelleted particles based on buoyant density and analyzed the different gradient fractions for infectivity. Based on densities reported above for naked virions and EV we subdivided the gradient in three segments with density ranges 1.15–1.35 g/ml, 1.06–1.13 g/ml, and 1.02–1.04 g/ml. In 10K-pelleted material, nearly all infectivity (87 ± 9.4%) was observed in the density fractions where EV reside (Fig 2A). For the 100K-pelleted material, the majority of the infectious particles resided in 1.15–1.35 g/ml density fractions, indicative of the presence of naked virus particles. Interestingly, however, infectivity (11 ± 5.7%) was also present in fractions with densities of 1.06–1.13 g/ml, characteristic for EV (Fig 2B). To further confirm that the infectious 10K and 100K particles in these fractions represented virus-containing EV, the presence of genomic viral RNA and the EV-associated tetraspanin proteins CD63 and CD9 [29–31] were assessed. Indeed, the viral RNA co-fractionated with CD63 and CD9 in the 1.06–1.13 g/ml density fractions. In contrast, high density fractions contained viral RNA but were devoid of CD63 and CD9, indicating the presence of naked virus (Fig 2C–2F). Within the 1.06–1.13 g/ml density range, we observed slightly different distributions of CD9, CD63 and viral RNA over the individual density fractions. This corresponds to previously published work indicating that different EV subpopulations are enriched in different tetraspanin proteins [32]. Moreover, the data could indicate that viral RNA is present in specific EV subpopulations. Detection of histon protein H3 was included to confirm that no contamination with intracellular protein complexes through cell lysis or cell death had occurred. Although readily detected in cell lysates, histon H3 was indeed absent from extracellular 10K and 100K centrifugation pellets (Fig 2D and 2F). In addition, we disrupted 10K and 100K EV by treatment with 0.1% triton ([33] and S3 Fig), prior to isopycnic density gradient centrifugation. This treatment depleted infectivity in the 1.06–1.13 g/ml fractions of the 10K and 100K pelleted material (Fig 2G and 2H). The infectivity lost in these fractions shifted to the high density segment of the gradient where naked virions reside, supporting the notion that EV in the 1.06–1.13 g/ml fractions contained mature virus particles.

To further confirm that EMCV was enclosed in EV, we compared the sensitivity of EV-enclosed viruses and naked viruses to neutralization by anti-EMCV capsid antibodies (Fig 2I). Protection of EV-enclosed viruses against neutralizing antibodies was first described for HAV by the group of S. Lemon [4]. Additionally, they showed that neutralizing antibodies could partially inhibit eHAV, but only after endocytosis of eHAV and degradation of the EV membranes. It is possible that co-endocytosed antibodies neutralize the virus upon degradation of the EV membrane, preventing interaction of the virus with viral receptors present in endosomes [21]. Partial neutralization of EV-enclosed virus has also been observed for EV71 [7]. Similarly, we observed that EV enclosure significantly protected EMCV against neutralizing antibodies, while the infectivity of naked virus could be fully abolished by this antibody (Fig 2I). Combined, these data indicate that infectious virus is released in both 10K and 100K EV populations during the pre-lytic phase of EMCV infection.

### 10K and 100K virus-containing EV differ in protein composition and release dynamics

**Fig 3:**
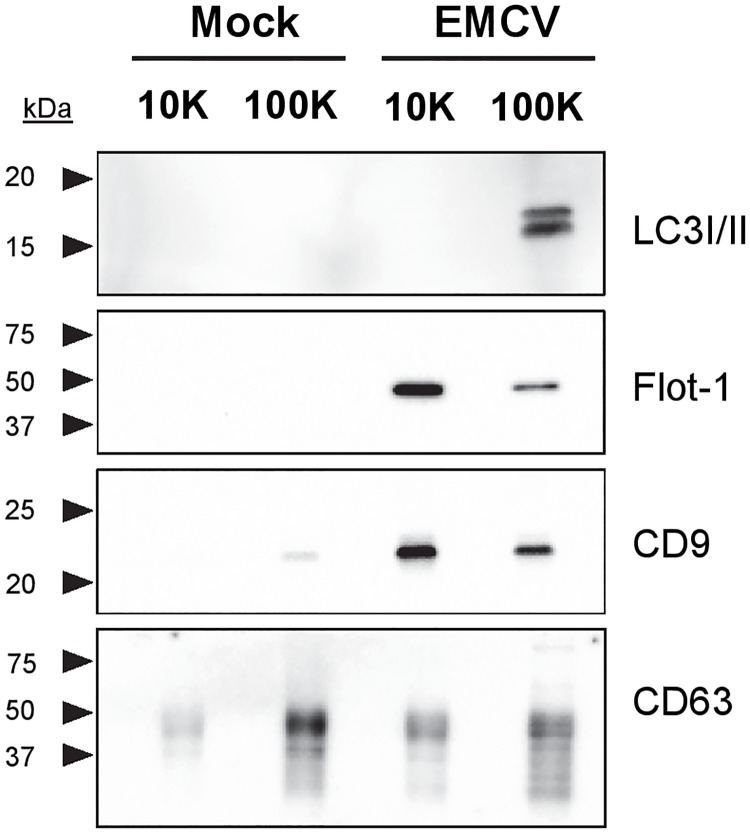
10K and 100K EV released by EMCV-infected cells differ in protein composition. 10K and 100K EV released by mock-infected and EMCV-infected cells 8 hrs p.i. were analyzed by western blotting for LC3, Flotillin-1, CD9 and CD63. Data are representative for three independent experiments.

To further assess differences in molecular composition between 10K and 100K EV released by virus-infected cells and mock cells, we performed western blot analysis for several proteins previously found to be associated with virus-containing EV. First we analyzed the presence of LC3, an autophagy regulatory protein previously detected on EV induced by picornaviruses of the *Enterovirus* genus [1–3,34]. LC3 was found prominently in EV pelleted at 100K, but not in 10K EV and EV released by mock cells (Fig 3). In contrast, virus-induced 10K EV were enriched in CD9 and Flotillin-1, proteins frequently associated with EV [11,22,32,35] (Fig 3). No clear differences were found between EV-associated levels of the tetraspanin CD63 in 10K and 100K EV from infected and mock cells (Fig 3). Higher levels of CD9 and Flotillin-1 were observed in EV fractions from infected cells compared to EV from mock cells. Overall, these data indicate that EMCV infection affects EV release and that 10K and 100K EV induced by EMCV display prominent differences in protein composition.

**Fig 4:**
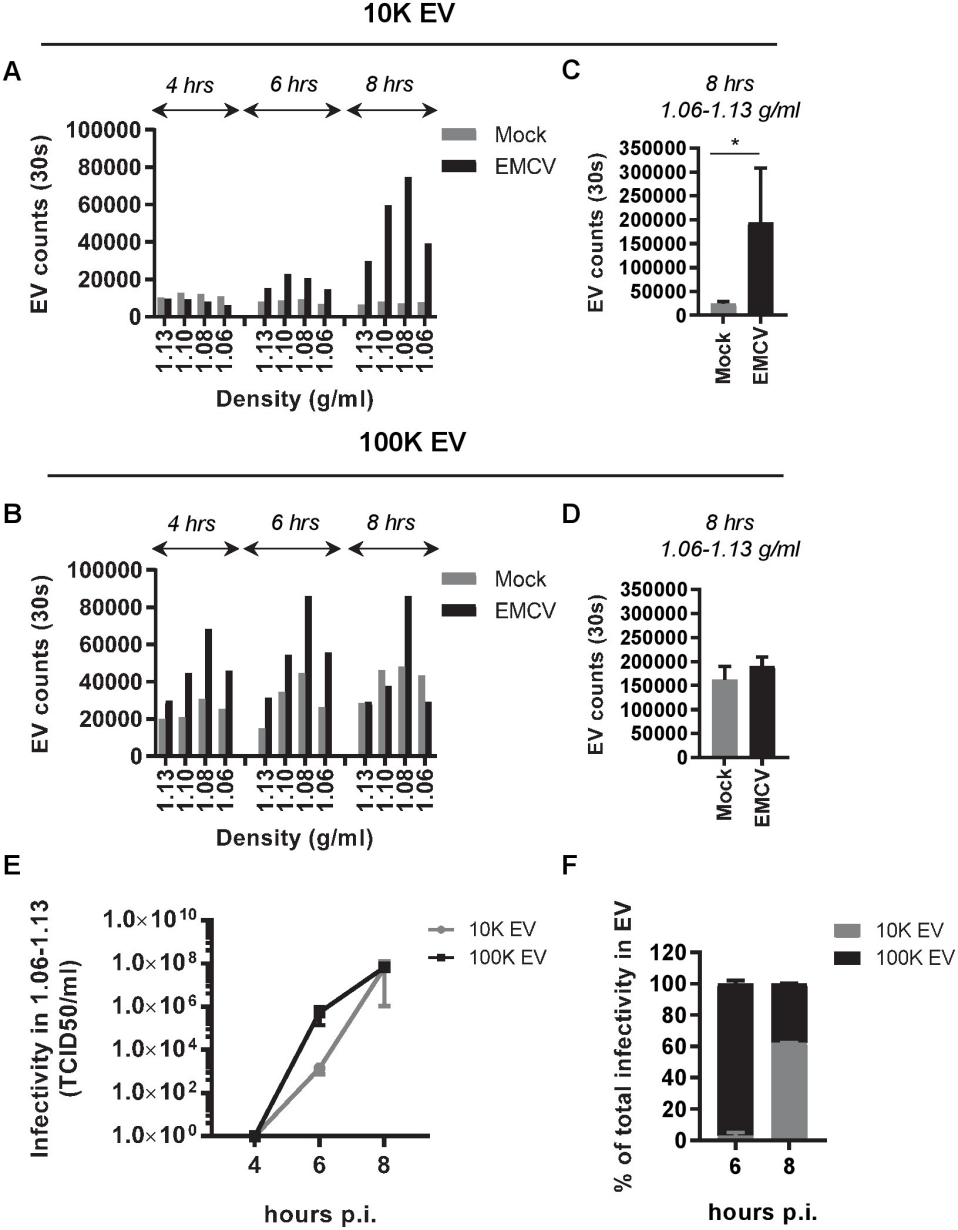
10K and 100K virus-containing EV are released by EMCV-infected cells at different time points after infection. (A-D): EV were fluorescently labeled with PKH67 and quantified by high-resolution flow cytometry. EV numbers were compared for virus-infected and mock-infected cells at 4, 6, and 8 hours after infection. Indicated are the number of EV acquired in a fixed time window of 30 seconds. (A,B): Bar graphs display the number of 10K (A) and 100K (B) EV in individual EV-containing density fractions at 4, 6, or 8 hrs p.i. Data are representative for N = 2 (10K EV (A)) and N = 3 (100K EV (B)) independent experiments. (C,D): Total numbers of 10K EV (C) and 100K EV (D) in density fractions (1.06–1.13 g/ml) released during 8 hrs of culture in Mock versus EMCV infected conditions (mean ± s.d. of N = 4 independent experiments, * p<0.05). (E) The infectivity in 10K and 100K EV fractions (1.06–1.13 g/ml) was assessed by end-point dilution at 4, 6 and 8 hours p.i. Indicated are mean values ± s.d. for N = 3 experiments. (F) The contribution of infectivity in 10K and 100K EV to total infectivity in EV was calculated at 6 and 8 hours p.i. Indicated are mean values and range of N = 2 independent experiments.

During the first 8 hours of infection, the cells undergo rapid changes in cell signaling and remodeling of intracellular membranes (reviewed in [36,37]). Since EV can be viewed as snapshots of their parental cell at the time of production, the composition and release of EV may change over the course of infection. We therefore analyzed the quantity and infectivity of virus-induced 10K and 100K EV at several time points during the pre-lytic phase of infection. For comparison, the release of 10K and 100K EV by non-infected cells cultured for the same period of time was analyzed in parallel. To assess quantitative differences between the EV, we employed an in-house developed high-resolution flow cytometry-based approach for high-throughput analysis of individual EV [23,24]. A fluorescent lipophilic dye was used to label the lipid bilayer of EV prior to density gradient ultracentrifugation. Fluorescence threshold triggering was applied to distinguish labeled EV from noise signals. EV quantification indicated a slight increase in the number of 10K EV released by infected cells from 4–6 hours p.i., and a much more prominent increase between 6–8 hours p.i. (Fig 4A). Non-infected cells, on the contrary, released only very few 10K EV. Substantially different release profiles were observed for 100K EV. Already at 4 hours p.i., considerable numbers of 100K EV were released from infected cells and these numbers gradually increased until 8 hours p.i. (Fig 4B). However, 100K EV released by non-infected cells followed a similar pattern. After 8 hours of infection, the cumulative number of 10K EV released by virus-infected cells was significantly higher than their counterparts released by non-infected cells, whereas comparable numbers of 100K EV were released by these cells (Fig 4C and 4D). In addition to EV quantification, we analyzed the infectivity of 10K and 100K EV fractions over the course of infection. At 4 hours p.i. no detectable levels of infectivity were present in 10K or 100K EV fractions (Fig 4E). At 6 hours p.i., infectious virus was primarily released in 100K EV, while the levels of infectivity in 10K EV were ~500-fold lower (Fig 4E and 4F). After 8 hours, similar levels of infectivity were detected in both the 10K EV and 100K EV (Fig 4E and 4F). These data demonstrate that the release of 100K EV-enclosed virus precedes the release of 10K EV-enclosed virus. Moreover, the combined EV quantification and infectivity data imply that there is temporal release of different EV subpopulations during the early phase of infection.

### Subsets of EV released by EMCV-infected cells differ in their efficiency to transfer infection

**Fig 5:**
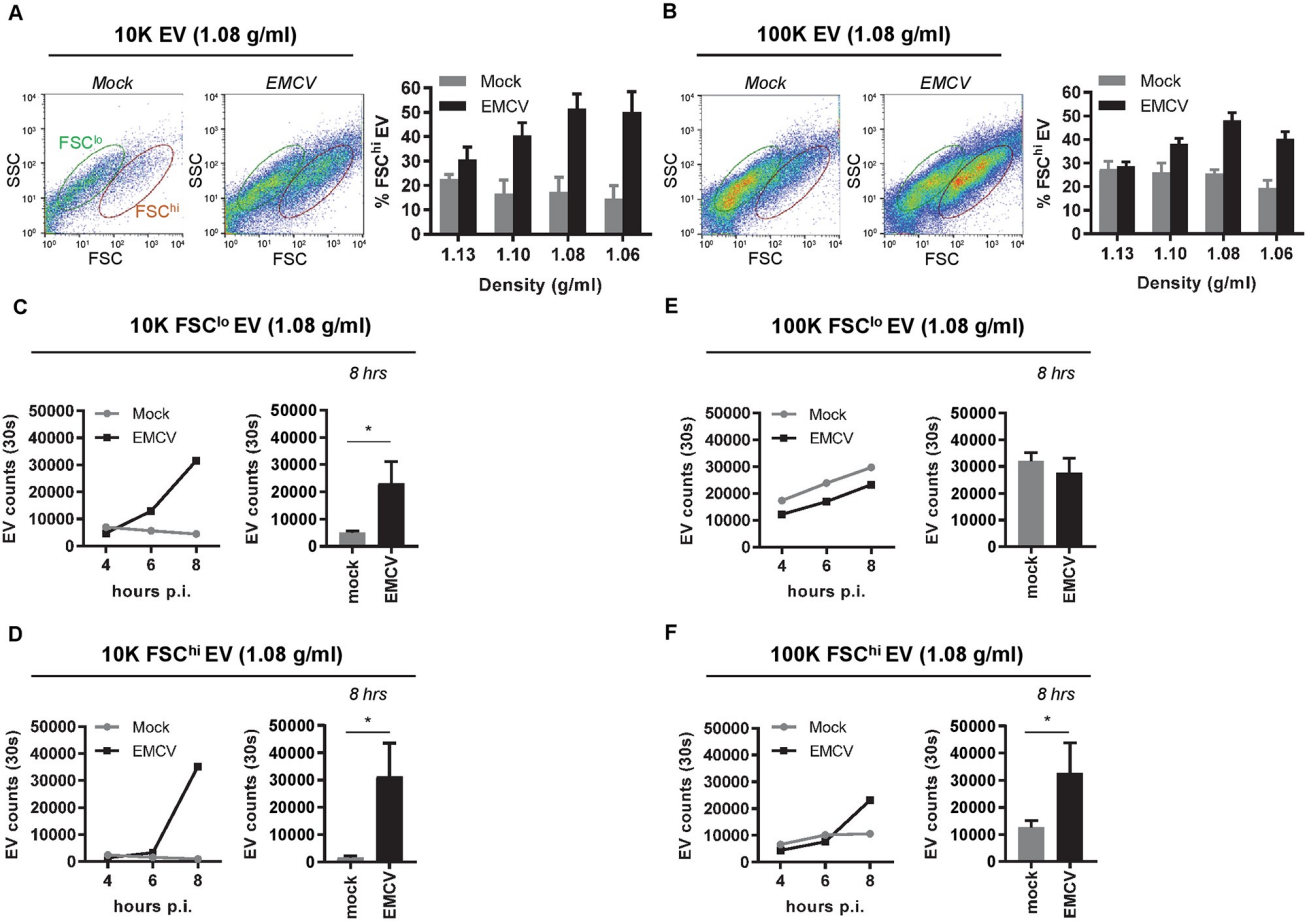
High-resolution flow cytometry reveals heterogeneity in light scattering properties of both 10K and 100K EV released during EMCV infection. (A,B): 10K (A) and 100K (B) EV from mock and EMCV-infected cells were labeled with PKH67 and analyzed by high-resolution flow cytometry. Depicted are representative FSC-SSC dot plots of EV in the 1.08 g/ml density fractions at 8 hours p.i. and gates indicate FSC^hi^ EV (red) and FSC^lo^ EV (green)(left panels). Bar graphs display the percentage of FSC^hi^ EV in 1.06–1.13 g/ml density gradient fractions in mock versus infected conditions (right panels). (C-F): EV counts were compared for FCS^lo^ (C,E) and FSC^hi^ (D,F) subpopulations of 10K (C,D) and 100K (E,F) EV (1.08 g/ml) released by mock and EMCV-infected cells. Indicated are EV numbers acquired in 30 seconds at 4, 6, and 8 hours p.i. Data in line graphs (left panels) are representative for N = 2 (10K EV) or N = 3 (100K) independent experiments. Bar graphs (right panels) indicate the number of FCS^lo^ and FSC^hi^ EV in mock versus infected conditions at 8 hrs of culture (mean number of EV acquired in 30 seconds ± s.d. for N = 3 experiments, * p<0.05).

Next, we investigated whether EV subpopulations with different infection potential exist within the population of EV released by infected cells. During high-resolution flow cytometric analysis of EV, we observed the presence of two distinct EV subpopulations among 10K and 100K EV released at 8 hours p.i. (Fig 5A and 5B). These two EV populations overlapped in the levels of side scattered light (SSC) but differed in the levels of forward scattered light (FSC) they induced, which is likely caused by differences in EV size and/or composition [38]. Interestingly, EV with high level FSC (FSC^hi^ EV) accounted for a much larger portion of the total EV population under infected *versus* non-infected conditions (Fig 5A and 5B).

First, we investigated whether the FSC^hi^ EV could represent membranous fragments from a small number of dead/lysed cells that could be present 8 hrs p.i. (see Fig 1). We addressed the contribution of 10% cell lysis to the number and type of EV observed in the supernatant of infected cells (S4 Fig). Supernatant of infected cells lysed by freeze-thawing (S4E Fig) was mixed with supernatant of healthy cells (containing mock EV) in a volume ratio of 10:90. EV populations were isolated, after which the quantity and light scatter profile of these EV were analyzed by high-resolution flow cytometry. The data clearly indicate that the large increase in both the total numbers of 10K EV (low and high FSC) and in the percentage of FSC^hi^ 100K EV released upon virus infection cannot be explained by contaminating material from lysed cells (S4A–S4D Fig). To further characterize FSC^hi^ and FSC^lo^ EV populations, we analyzed the presence of the common EV marker CD9 on individual FSC^lo^ and FSC^hi^ EV using high-resolution flow cytometry (S5 Fig). These data indicate that CD9 was a common marker of the various EV populations detected, but that the level of CD9 on FSC^hi^ EV was generally higher than on FSC^lo^ EV.

Next, the release of the different EV subpopulations was monitored over time. We observed a sharp increase in both 10K FSC^hi^ and 10K FSC^lo^ EV released by infected cells during the 6–8 hour p.i. interval (Fig 5C and 5D). This suggests that both of these EV subsets are induced by virus infection. In the 100K EV population, on the contrary, only the number of FSC^hi^ EV was significantly increased upon virus infection (Fig 5E and 5F). The number of FSC^lo^ EV released by infected cells increased over time to the same extent as FSC^lo^ EV released by non-infected cells, suggesting that this population represents constitutively released EV. The observed temporal differences in release of these EV subpopulations may indicate that they play a distinct role during infection.

**Fig 6:**
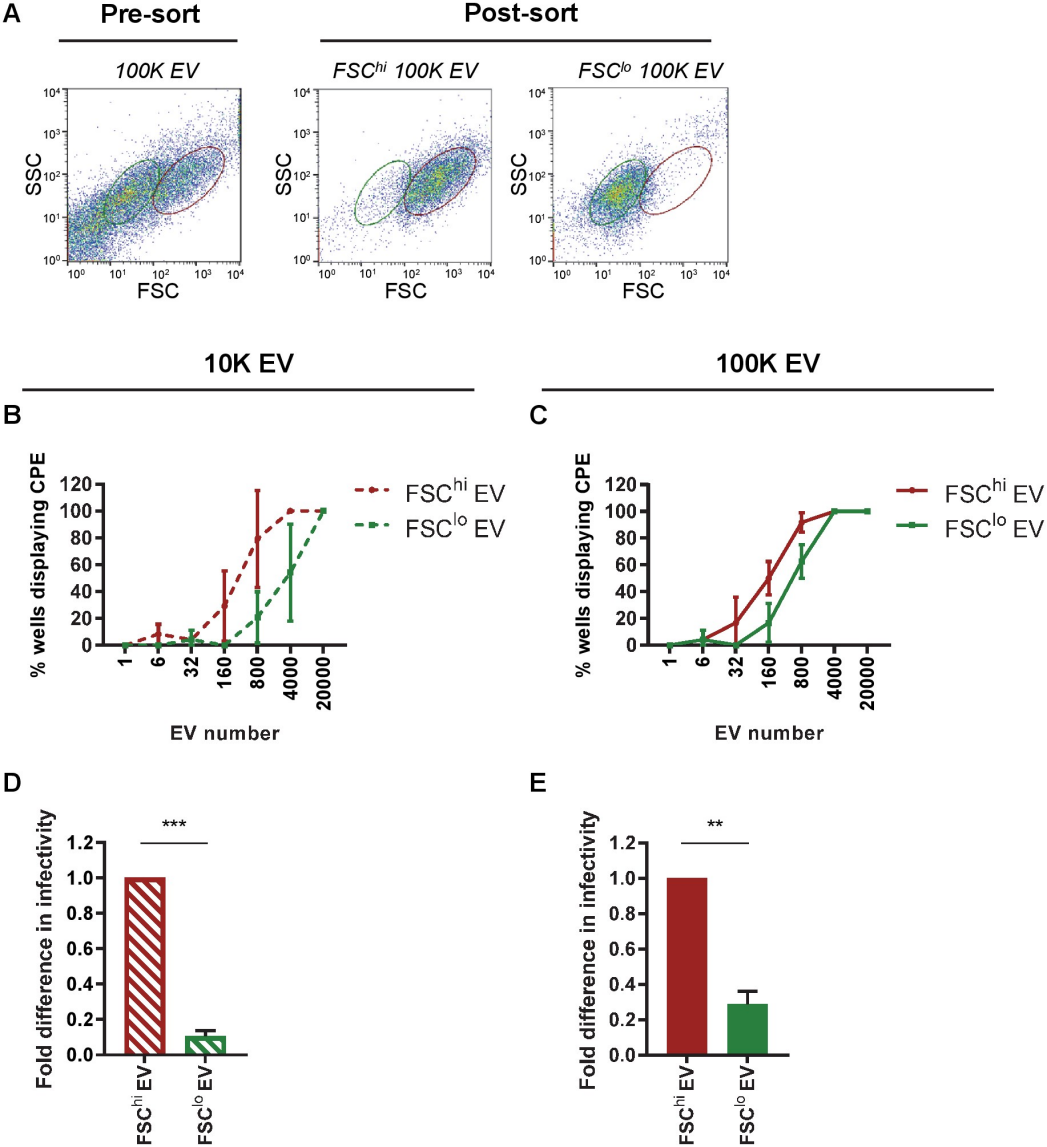
EV subsets distinguished based on light scattering properties differ in potency to transfer virus-infection. 10K and 100K EV (1.08 g/ml) from EMCV-infected cells were labeled with PKH67 and analyzed by high-resolution flow cytometry. PKH67-positive EV with different FSC profiles (FSC^hi^ and FSC^lo^) were identified and isolated by flow cytometric sorting. (A) Depicted are representative FSC-SSC dot plots of 100K EV before and after sorting of the indicated populations (green = FSC^lo^ EV, red = FSC^hi^ EV). (B-C): For both 10K (B) and 100K (C) EV populations, the indicated numbers of FSC^hi^ EV and FSC^lo^ EV were sorted directly onto recipient cells in 96-wells plates, after which wells were screened for occurrence of CPE. Depicted are the percentages of wells displaying CPE upon addition of the indicated numbers of FSC^hi^ and FSC^lo^ EV. Bar graphs (D,E) show relative differences in infectivity between FSC^hi^ and FSC^lo^ EV for both 10K (D) and 100K (E) EV, with the infectivity in FSC^hi^ EV set to 1. Indicated are mean values ± s.d. for N = 3 independent experiments (** p<0.005, *** p<0.0005).

Since EMCV infection most prominently increased the release of FSC^hi^ EV, we next investigated whether these EV were functionally different from FSC^lo^ EV. We applied a unique approach using high-resolution flow cytometric sorting of single EV [39] to physically separate FSC^hi^ and FSC^lo^ EV subsets. With this method we reached ~90% sort purity for the different EV subpopulations (Fig 6A and Materials & methods). To quantitatively compare the efficiency of virus transfer by FSC^hi^ and FSC^lo^ EV populations, defined numbers of EV (range 1–20,000) were sorted directly onto recipient cells in culture. Cells and EV were incubated for 3 days after which the occurrence of virus-induced cytopathogenic effect (CPE) was assessed. Interestingly, for some EV subpopulations as few as 6–32 sorted EV could cause infection of recipient cells. We compared the percentage of wells displaying CPE after incubation with different numbers of FSC^hi^ or FSC^lo^ EV and observed substantial differences in infectious potential between EV subpopulations (Fig 6B and 6C). Based on the calculated 50% tissue culture infectivity dose (TCID_50_), we show that both FSC^hi^ 10K EV and FSC^hi^ 100K EV were significantly more potent in transferring infection than their FSC^lo^ counterparts (Fig 6D and 6E). CPE in EV-recipient cells was confirmed to be caused by replicating virus by RT-qPCR analysis for viral genomic RNA in the affected cells (S6A and S6B Fig). Together, these results show that EMCV-infected cells release various subsets of EV which differ in molecular composition and infection potential and that these distinct EV subsets are released at different time points after infection.

## Discussion

To our knowledge, our data provide first evidence that a picornavirus from the *Cardiovirus* genus, like other members of the Picornaviridae family, can exit cells via enclosure in EV. Using advanced single EV-based analysis and isolation methods, we demonstrated that host cells respond to EMCV infection by releasing a complex mixture of EV that are heterogeneous in their molecular composition and their capacity to transfer viral infection. In addition, we demonstrate differences in the release kinetics of these distinct EV subpopulations during the pre-lytic phase of infection.

Importantly, this study demonstrates that naked viruses can be released by infected cells in multiple subtypes of EV, which may form via different biogenesis pathways and perform different functions during infection. The distinct virus-containing EV populations released during EMCV infection differed in sedimentation efficiency, which could signify a difference in size [26–28]. Although the differential (ultra)centrifugation method applied in our study does not allow complete separation of large (10K) and small (100K) EV, clear differences in protein composition were observed, with 10K EV bearing high levels of the plasma membrane resident proteins CD9 and Flotillin-1, and 100K EV prominently containing the autophagy regulatory protein LC3. Previous studies on EV-mediated naked virus release may have failed to recognize this heterogeneity among the virus-containing EV, because large 10K EV were either not separated from small 100K EV during sample preparation or omitted from analysis. In our recent review on methodologies to isolate naked virus-containing EV, we highlight the large diversity in methods that have been used in previously published studies on this subject [15]. Since these methods differ in yield and purity of EV and bias towards isolating specific EV subpopulations, this can greatly influence findings on the molecular composition and function of EV and can hamper data comparison between studies. Our data additionally indicate that virus-containing subpopulations can differ in release kinetics. Collection of EV during different phases of infection may therefore further hamper data comparability between studies. These findings stress the need to address temporal and compositional heterogeneity of EV populations induced by virus infection.

Heterogeneity in EV populations may arise from multiple biogenesis routes driving concurrent release of EV with different subcellular origin and different molecular composition. Since proteins involved in EV formation are also incorporated in EV, detection of such proteins in EV preparations could be indicative of their biogenesis route. The presence of LC3 on 100K EV released by infected cells could suggest the involvement of autophagosomal membranes in the formation of these virus-containing EV, as was described earlier for picornavirus species of the *Enterovirus* genus [1,2,34]. Similar to poliovirus, CVB3, and EV71, EMCV has been shown to induce autophagy in host cells [40–42]. Conversely, 10K EV released by infected cells were enriched in Flotillin-1, a caveolae-associated membrane protein, and CD9, a tetraspanin ubiquitously reported on EV originating from various cellular membranes [30,32]. CD63, a tetraspanin more specifically associated with MVB-derived EV [30–32], was detected at similar levels on 10K and 100K EV. The abundant presence of Flotillin-1 and CD9 without a corresponding enrichment in CD63 may imply that the 10K EV are not formed in MVBs but have a different subcellular origin, and could represent plasma membrane derived EV.

An additional source of heterogeneity within EV populations stems from the rapid and drastic morphological and metabolic changes that the EV-producing cells undergo over the course of the infection. Because EV are snapshots of their parental cell at the moment of production, over time this can lead to accumulation of EV with different molecular composition. In support of this idea, we showed that the composition and release of EV changes over the course of EMCV infection. This could imply that multiple EV formation pathways are active at distinct time points after infection. The sequential employment of different EV-formation routes may reflect virus-driven shut-down or activation of these pathways. Alternatively, it may be a coping mechanism of the host cell to deal with the accumulating intracellular viral burden. Upon sensing invading viruses, the host cell activates antiviral systems and the accompanying cellular signaling cascade may alter the release and composition of EV.

In-depth characterization of the compositional and functional heterogeneity of EV populations is lagging behind due to the lack of high-end technologies to analyze EV at the single particle level. We used our in-house developed high-resolution flow cytometric approach to detect and characterize individual EV. Using this technique we were able to distinguish as well as physically separate EV subpopulations that differ in their potential to transfer infection. We were able to discriminate these EV populations based on differences in the level of FSC light they generated. Due to the overlapping size and buoyant density of these EV subsets, they cannot be separated with any of the other frequently used EV isolation methods. FSC^lo^ EV were much less potent in transferring virus infection to new cells than FSC^hi^ EV. The differences in infection potential between these EV subsets may be explained in multiple ways. First, the proportion of virus-containing EV within the FSC^lo^ EV population may be smaller than in the FSC^hi^ EV population. Alternatively, FSC^hi^ EV may enclose a higher number of virus particles than FSC^lo^ EV and therefore promote infection, for example by facilitating cooperativity between virus genetic quasispecies [1,43,44]. Secondly, since very small numbers of sorted FSC^hi^ EV could drive infection of EV-recipient cells and 100% sort purity cannot be reached due to technical limitations, the observed infectivity in FSC^lo^ EV may be caused by a minor contamination with FSC^hi^ EV. Finally, it is possible that both FSC^hi^ and FSC^lo^ EV contain virus particles but that different sets of host molecules incorporated in these EV aid or restrict the entry of the virus or in establishing infection. The number of low infectivity FSC^lo^ 10K EV was highly increased upon virus infection, suggesting that these EV could play a modulatory role in promoting or counteracting viral infection. For example, the EV-mediated transfer of various host antiviral molecules from virus-infected cells has been described previously [45–47]. Whether the EV with different infection potential in our study carry distinct proteins, lipids, or RNA markers based on which they could be distinguished needs to be explored in future studies.

Overall, this study shows that naked virus-infected cells release multiple EV subpopulations that differ in physical properties, molecular content, and function. Additionally, we demonstrate that temporal release of the different subpopulations of EV is tightly regulated over the course of infection. This heterogeneity in EV populations may underlie the previously observed diversity in functional properties of EV released by naked virus-infected cells. In-depth characterization of the compositional, functional and temporal heterogeneity of EV released over the course of infection is essential to delineate the relationship between the structure of these EV and their function in virus spreading and host responses.

## Materials and methods

### Cells and virus

Human cervical carcinoma cells (HeLa R19) were a gift from Dr G. Belov (University of Maryland, USA) and baby hamster kidney cells (BHK21, ATTC CCL-10) were obtained from the American Type Culture Collection (Rockville, MD). Cell lines were maintained in Iscove’s modified Dulbecco’s medium (IMDM; Lonza, Basel, Switzerland), supplemented with 10% fetal calf serum (FCS; GE Healthcare Bio-Sciences, Chicago, IL), 2 mM Ultraglutamine (Lonza), 100 U mL^-1^ penicillin and 100 μg mL^-1^ streptomycin (Gibco, Paisley, United Kingdom), in a humidified incubator at 37°C in an atmosphere with 5% CO_2_.

EMCV stocks were obtained by transfection of BHK21 cells with *in vitro* RNA transcripts of the previously described infectious cDNA clone pM16.1, which contains a copy of the EMCV genome with a shortened poly-C tract [48]. Virus was harvested after observing virus-induced CPE and thereafter concentrated from cell-free culture supernatants by high-speed ultracentrifugation through a 30% sucrose cushion at 80,000x*g* for 16 hrs in a SW32 rotor (*k*-factor 321) (Beckman Coulter, Brea, CA).

### Cell viability and membrane integrity assays

To assess cell viability and membrane integrity after EMCV infection, HeLa R19 cells were seeded at 1.5 x 10^5^ mL^-1^ in 24-wells plates and infected the next day at multiplicity of infection (MOI) = 10. Unbound virus was removed 1 hr post-infection (p.i.) by washing cells 3 times with phosphate buffered saline (PBS). Cells and supernatant were harvested at 2 hr intervals until 10 hrs p.i. and at 20 hrs p.i. Cell viability was assessed using Fixable Viability Dye eFluor780 (eBioscience, San Diego, CA) according to the manufacturer’s protocol. In short, cells were harvested, washed with PBS, and stained with 1 μL dye per 1x10^6^ cells for 30 min at 4°C, washed with PBS, and fixed in 1% paraformaldehyde (PFA). Cells were analyzed using a BD FACS Canto II (BD Biosciences, San Jose, CA) with BD FACS Diva software. Cell membrane integrity was assessed using the CytoTox 96 Non-Radioactive Cytotoxicity Assay (Promega, Madison, WI) according to the manufacturer’s guidelines. The presence of leaked cytoplasmic enzyme LDH in cell culture supernatants was determined after enzymatic conversion and absorbance was measured at 490 nm in a 96-well plate reader.

### Purification of EV

For isolation of EV from pre-lytic EMCV-infected cell cultures, HeLa R19 cells were seeded at ± 3 x 10^5^ mL^-1^ in T225 culture flasks and infected the next day at MOI = 10. Unbound virus was removed at 1 hr p.i. by washing the cells 3 times with PBS. Cells were then cultured in culture medium containing EV-depleted FCS. To remove EV from FCS, 30% FCS in IMDM was ultracentrifuged for 16–20 hrs at 100,000x*g* in an SW32 rotor (*k*-factor 256.8) and passed through a 0.22μm filter. EV-containing culture supernatant was collected at 8 hrs p.i. unless specified otherwise and sequentially centrifuged at 2x 200x*g* for 10 min and 2x 500x*g* for 10 min. 10K EV were isolated by ultracentrifugation of 500x*g* supernatant at 10,000x*g* for 30 min (*k*-factor 2567.7), and 100K EV were isolated by ultracentrifugation of 10,000x*g* supernatant at 100,000x*g* for 65 min in a SW32 rotor. EV-containing 10K and 100K ultracentrifugation pellets were resuspended in 20 μL PBS + 0.2% BSA (cleared from aggregates by ultracentrifugation for 16–20 hrs at 100,000x*g*) for high-resolution flow cytometry or in 300 ul PBS + 0.2% BSA for RNA isolation or western blotting. Resuspended EV pellets were mixed with 60% iodixanol (Optiprep; Axis-Shield, Oslo, Norway) to a final concentration of 45% iodixanol and overlaid with a linear gradient of 40%-5% iodixanol in PBS. Density gradients were centrifuged at 192,000x*g* for 15–18 hrs in a SW40 rotor (*k*-factor 144.5) (Beckman-Coulter). Gradient fractions of 1 mL were collected and densities were determined by refractometry.

### High-resolution flow cytometry

For high-resolution flow cytometric analysis of EV, 10K and 100K pelleted EV were labeled with 1.5 μl PKH67 (Sigma-Aldrich, St. Louis, MO) in 200 μl Diluent C. For detection of CD9 on single EV, antibody-labeling was performed prior to generic PKH67 labeling. 10K and 100K pelleted EV were labeled with 20 ng of PE-conjugated mouse-α-CD9 (clone HI9a; Biolegend, San Diego, CA) or a matched isotype control for 1 hr at 4°C. Unbound antibodies and unbound dye were separated from EV by density gradient centrifugation as described above. EV in gradient fractions were fixed with 2% paraformaldehyde for 30 min and diluted 20x in PBS for high-resolution flow cytometric analysis on a BD Influx flow cytometer with optimized configuration, as previously described in detail [23,24]. In short, threshold triggering was applied on fluorescence derived from PKH67-labeled EV passing the 488 nm laser. The threshold level was set to allow an event rate of ≤ 10 events per second when measuring PBS. Fluorescence of the 488 nm laser, and forward (FSC) and side (SSC) scattered light were recorded. FSC was detected with a collection angle of 15–25° (reduced wide-angle FSC). Fluorescent 100 nm and 200 nm polystyrene beads (FluoSpheres, Invitrogen, Carlsbad, CA) were used to calibrate the fluorescence and rw-FSC settings. Samples were measured at low pressure (5 PSI on the sheath fluid and 4.2 PSI on the sample) using a 140 μm nozzle at event rates below 10,000 per second. All measurements were acquired in a fixed time window of 30 seconds to allow direct comparison of EV concentrations in parallel samples. Data analysis was performed using FlowJo software (FlowJo LLC, Ashland, OR).

### High-resolution flow cytometric single EV sorting

For sorting of FSC^hi^ and FSC^lo^ EV, hardware adaptations and configuration adjustments on the BD Influx flow cytometer were employed to allow for maximal event rates and minimal sort volumes [39]. An 8 mm obscuration bar and 200 μm pinhole were placed on the FSC detector. In addition, a 70 μm nozzle was used, sheath fluid pressure was increased to 30 psi and sample fluid pressure was raised to reach a maximum event rate of ≤ 10,000 events per second. Sort efficiencies remained high (~95%) at a drop frequency of 67.3 kHz. Sort purity was confirmed upon re-analyzing sorted samples and reached 76 ± 3.7% and 84 ± 13.0% for 10K and 100K FSC^hi^ EV respectively, and 90 ± 1.9% and 88 ± 3.1% for 10K and 100K FSC^lo^ EV respectively. Different numbers (1–20,000) of FSC^hi^ and FSC^lo^ EV subpopulations were sorted directly in 8-fold replicates onto HeLa R19 cells in 96-wells clusters seeded a day before at 5.0 x 10^4^ cells mL^-1^. Three days after sorting, virus-induced CPE was observed and infectivity of EV subpopulations was calculated using the Spearman-Karber calculation method.

### Neutralization assay

1.08 g/ml (EV-virus) and 1.21 g/ml (naked virus) 100K density gradient fractions were diluted to 1*10^6 TCID_50_/ml and incubated with polyclonal anti-EMCV capsid antibody (kindly gifted by A. Palmenberg) in various dilutions (1:1000, 1:4000 and 1:16000) or an equal amount of PBS for 1h at RT. Antibody-EV/virus mixes were subsequently used for end-point dilution to determine infectivity.

### End-point dilution

Intracellular infectivity levels were assessed by subjecting infected HeLa R19 cells to 3 consecutive freeze-thaw cycles. Supernatants cleared from cell debris by centrifugation were used either directly or after the indicated centrifugation steps for determination of extracellular infectivity. Infectivity in high-grade purified EV was assessed by sampling directly from EV-containing density gradient fractions. HeLa R19 cells in 96-well clusters were infected with 3-fold serial dilutions of the above described material, and TCID_50_ values were calculated 3 days after infection using the Spearman-Karber calculation method.

### RNA isolation and RT-qPCR

Viral RNA was isolated from density gradient fractions using the Nucleospin RNA virus kit (Macherey Nagel, Düren, Germany) according to the manufacturers’ protocols. CVB3 virus was spiked into density gradient samples prior to RNA isolation as an internal reference control. cDNA was synthesized using random hexamer priming and TaqMan reverse transcription reagents (Applied Biosystems, Foster city, CA) according to the provided protocol. Quantitative analysis of EMCV and CVB3 genomic RNA levels in gradient fractions was performed on a LightCycler 480 (Roche, Basel, Switzerland) using SYBR Green master mix (Roche) and data analysis was performed with the provided software.

To confirm virus production 3 days after addition of sort-purified EV subsets, healthy cells and cells displaying CPE were subjected to 3 consecutive freeze/thaw cycles. Total cellular RNA in supernatants was isolated using the miRNeasy microkit (Qiagen, Hilden, Germany) according to the manufacturers’ instructions. cDNA was synthesized using the RevertAid First strand cDNA synthesis kit (ThermoFisher Scientific, Germany) using random hexamers according to the manufacturers’ guidelines. Quantitative analysis of EMCV genomic RNA in cells was performed on a Bio-Rad iQ5 Multicolor Real-Time PCR Detection System (Bio-Rad, Hercules, CA) using SYBR Green Sensimix (Bioline Reagents Ltd., London, United Kingdom). Quantification cycle (Cq) values were determined using BioRad CFX software.

The following primers were used: EMCV forward: 5’-TCTGTTCTGCCTGCTGTTTG-3’, EMCV reverse: 5’-AAAGAAGAGGGTGCCGAAAT-3’, CVB3 forward: 5’-CGTGGGGCTACAATCAAGTT-3’, and CVB3 reverse: 5’-TAACAGGAGCTTTGGGCATC3’.

### SDS-PAGE and western blotting

Gradient-purified EV were collected from individual or pooled density fractions (1.06–1.13 g/ml), diluted in PBS + 0.1% BSA and centrifuged at 192,000x*g* for 90 min in an SW40 rotor for individual gradient fractions and at 100,000x*g* for 90 min in an SW32 rotor for pooled fractions. For analysis of control cell lysates, cells were lysed in RIPA buffer (40 mM Tris-Hcl pH 8, 0.5% sodium deoxycholate, 1% Triton X-100, 150 mM sodium chloride, 0.1% sodium dodecyl sulfate) with a protease inhibitor cocktail (Roche). Lysates were cleared by centrifugation at 16,000×g for 15 min and protein concentration was determined by Pierce BCA assay kit (ThermoScientific, Waltham, MA) according to the manufacturer’s instructions. Purified EV, cell lysates, or 10K and 100K ultracentrifugation pellets were denatured at 100°C for 4 min in non-reducing Laemmli sample buffer (LSB) for detection of CD9 and CD63, or reducing LSB for detection of LC3 and Histone H3. Proteins were separated on 12.5% sodium dodecyl sulfate-polyacrylamide gels by electrophoresis (SDS-PAGE) and transferred to Immobilon 0.20 μM (LC3) or 0.45 μM (Flotillin-1, CD9, CD63, histone H3) PVDF membranes (Merck Millipore Ltd., Cork, Ireland) by wet transfer. Membranes were incubated with blocking buffer (0.2% fish skin gelatin (FSG; Sigma-Aldrich) + 0.1% Tween 20 in PBS) for 1 hr. Membranes were incubated for >16 hrs at 4°C with the following primary antibodies: mouse-α-CD63 (1:1000, clone TS63; Abcam, Cambridge, United Kingdom), mouse-α-CD9 (1:2000, clone HI9a; Biolegend), mouse-α-LC3 (1:500, clone 5F10; Nanotools, Teningen, Germany), mouse-α-Flotillin-1 (1:1000, clone 18/Flotillin-1; BD Biosciences), and polyclonal rabbit-α-Histone H3 (1:1000; Cell Signaling Technology, Danvers, MA) diluted in blocking buffer. Membranes were subsequently incubated for 1 hr with HRP-coupled secondary antibodies goat-anti-mouse (1:10,000; Jackson ImmunoResearch Labaratories Inc., West Grove, PA) or goat-anti-rabbit (1:1000; Agilent Technologies Inc., Santa Clara, CA), diluted in blocking buffer. In between primary and secondary antibody incubations membranes were washed at least 5 times with blocking buffer. After secondary antibody incubations membranes were washed 5 times with 0.1% Tween-20 in PBS and additional 3 times with PBS. ECL solution (SuperSignal West Dura Extended Duration Substrate, ThermoScientific) was used for detection on a Bio-Rad ChemiDoc imaging system and images were analyzed by Image Lab software (Bio-Rad).

## Supporting information

S1 Fig Cell integrity at different time points after infection. Flow cytometric analysis of mock cells and cells infected with EMCV stained with fixable Viability Dye eFluor780 after culture for the indicated amount of time. Heat killed cells were analyzed as a positive control, based on which a gate was set for eFluor780-positive events. For each condition, at least 20,000 events were recorded and the percentages of dead cells are indicated. Presented dot plots are representative of three experiments.
(TIF)

S2 Fig EMCV naked virions and EV differ in buoyant density. (A) EMCV virus particles harvested during the lytic phase of infection were treated with 0.1% triton to disrupt residual lipid membranes prior to buoyant density gradient centrifugation. Depicted is the infectivity in individual gradient fractions assessed by end-point dilution. (B) 100K EV from non-infected cells were separated on buoyant density gradients. Individual gradient fractions and control whole cell lysates (WCL) were analyzed for the presence of EV marker protein CD63 by western blotting. Presented are representative data of two independent experiments for A and B.
(TIF)

S3 Fig EV are disrupted by treatment with 0.1% triton. Efficiency of disruption of PKH67-labeled EV by treatment with 0.1% triton was assessed by high-resolution flow cytometry. Depicted are representative dot plots of control EV, triton-treated EV, or background events (PBS) detected above the fluorescence threshold during a 30 seconds acquisition.
(TIF)

S4 Fig Increased number of EV released upon EMCV infection cannot be explained by contaminating material from lysed cells. (A, B) 10K (A) and 100K (B) EV were isolated from supernatants of mock cells (left), EMCV-infected cells 8 hrs p.i. (middle), and mixed supernatants of lysed infected cells (10 v/v%) and mock cells (90 v/v%). EV were labeled with PKH67 and analyzed by high resolution flow cytometry. FSC-SSC plots represent quantitative flow cytometric measurements (30 seconds fixed time window) of EV in the 1.08 g/ml density fraction. (C, D) Bar graphs display the total number of 10K EV acquired during the 30 seconds measurements (C) and the percentage of FSC^hi^ EV of the total 100K EV detected in the indicated conditions (D). (E) Lysis of cells by freeze/thaw cycling was confirmed to be complete and comparable to triton-mediated lysis of cells by measuring leakage of the intracellular enzyme LDH into the extracellular space. Data are representative for two independent experiments.
(TIF)

S5 Fig EV subpopulations released by EMCV-infected cells display different levels of CD9. High resolution flow cytometric analysis of 10K (A) and 100K (B) EV concurrently labeled with PKH67 and PE-conjugated anti-CD9 or isotype control antibodies. Indicated are histogram overlays (left) and geometric mean fluorescence intensities (right) for CD9 relative to a matched isotype control detected on single FSC^hi^ or FSC^lo^ EV.
(TIF)

S6 Fig CPE in EV-recipient cells is caused by virus replication. Viral genomic RNA levels in recipient cells of sort-purified EV subsets was assessed 3 days after sorting by RT-qPCR to confirm that the observed CPE was caused by EV-mediated transfer of infection and subsequent production of progeny virus. (A) Microscopic images showing recipient cells of EV that are healthy (left) or display CPE (right). Bar = 200 μm. (B) Cq values for viral genomic RNA in healthy cells that did not receive EV, healthy cells that received EV from mock-infected cells, and cells displaying CPE that received EV from EMCV-infected cells. Indicated are mean values ± s.d. for N = 3 independent experiments.
(TIF)

## References

[1] Chen YH, Du W, Hagemeijer MC, Takvorian PM, Pau C, Cali A, et al Phosphatidylserine vesicles enable efficient en bloc transmission of enteroviruses. Cell. 2015;160(4):619–30. 10.1016/j.cell.2015.01.032 25679758 PMC6704014

[2] Robinson SM, Tsueng G, Sin J, Mangale V, Rahawi S, McIntyre LL, et al Coxsackievirus B Exits the Host Cell in Shed Microvesicles Displaying Autophagosomal Markers. PLoS Pathog. 2014;10(4). 10.1371/journal.ppat.1004045 PMC3983045 24722773

[3] Too IHK, Yeo H, Sessions OM, Yan B, Libau EA, Howe JLC, et al Enterovirus 71 infection of motor neuron-like NSC-34 cells undergoes a non-lytic exit pathway. Sci Rep [Internet]. Nature Publishing Group; 2016;6(January):1–16. Available from: 10.1038/srep36983 27849036 PMC5111112

[4] Feng Z, Hensley L, McKnight KL, Hu F, Madden V, Ping L, et al A pathogenic picornavirus acquires an envelope by hijacking cellular membranes. Nature [Internet]. Nature Publishing Group; 2013;496(7445):367–71. Available from: 10.1038/nature12029 23542590 PMC3631468

[5] Colombo M, Raposo G, Théry C. Biogenesis, Secretion, and Intercellular Interactions of Exosomes and Other Extracellular Vesicles. Annu Rev Cell Dev Biol [Internet]. 2014;30(1):255–89. Available from: http://www.annualreviews.org/doi/10.1146/annurev-cellbio-101512-122326 25288114 10.1146/annurev-cellbio-101512-122326

[6] Yáñez-Mó M, Siljander PRM, Andreu Z, Zavec AB, Borràs FE, Buzas EI, et al Biological properties of extracellular vesicles and their physiological functions. J Extracell Vesicles. 2015;4(2015):1–60. 10.3402/jev.v4.27066 PMC4433489 25979354

[7] Mao L, Wu J, Shen L, Yang J, Chen J, Xu H. Enterovirus 71 transmission by exosomes establishes a productive infection in human neuroblastoma cells. Virus Genes. Springer US; 2016;52(2):189–94. 10.1007/s11262-016-1292-3 26837894

[8] Nagashima S, Jirintai S, Takahashi M, Kobayashi T, Tanggis, Nishizawa T, et al Hepatitis E virus egress depends on the exosomal pathway, with secretory exosomes derived from multivesicular bodies. J Gen Virol. 2014;95(2014):2166–75. 24970738 10.1099/vir.0.066910-0

[9] Takahashi M, Tanaka T, Takahashi H, Hoshino Y, Nagashima S, Jirintai, et al Hepatitis e virus (HEV) strains in serum samples can replicate efficiently in cultured cells despite the coexistence of HEV antibodies: Characterization of HEV virions in blood circulation. J Clin Microbiol. 2010;48(4):1112–25. 10.1128/JCM.02002-09 20107086 PMC2849599

[10] Takahashi M, Yamada K, Hoshino Y, Takahashi H, Ichiyama K, Tanaka T, et al Monoclonal antibodies raised against the ORF3 protein of hepatitis e virus (HEV) can capture HEV particles in culture supernatant and serum but not those in feces. Arch Virol. 2008;153(9):1703–13. 10.1007/s00705-008-0179-6 18679765

[11] Nagashima S, Takahashi M, Kobayashi T, Tanggis, Nishizawa T, Nishiyama T, et al The characterization of the quasi-enveloped hepatitis E virus particles released by the cellular exosomal pathway. J Virol [Internet]. 2017;91(22):JVI.00822-17. Available from: http://jvi.asm.org/lookup/doi/10.1128/JVI.00822-17 10.1128/JVI.00822-17 PMC5660490 28878075

[12] Chapuy-Regaud S, Dubois M, Plisson-Chastang C, Bonnefois T, Lhomme S, Bertrand-Michel J, et al Characterization of the lipid envelope of exosome encapsulated HEV particles protected from the immune response. Biochimie. 2017;141:70–9. 10.1016/j.biochi.2017.05.003 28483690

[13] Feng Z, Li Y, McKnight KL, Hensley L, Lanford RE, Walker CM, et al Human pDCs preferentially sense enveloped hepatitis A virions. J Clin Invest. 2015;125(1):169–76. 10.1172/JCI77527 25415438 PMC4382264

[14] Das A, Hirai-Yuki A, González-López O, Rhein B, Moller-Tank S, Brouillette R, et al TIM1 (HAVCR1) is not essential for cellular entry of either quasi-enveloped or naked hepatitis a virions. MBio. 2017;8(5):1–14. 10.1128/mBio.00969-17 PMC5587907 28874468

[15] van der Grein SG, Defourny KAY, Slot EFJ, Nolte-‘t Hoen ENM. Intricate relationships between naked viruses and extracellular vesicles in the crosstalk between pathogen and host. Semin Immunopathol [Internet]. Seminars in Immunopathology; 2018;1–14. Available from: http://link.springer.com/10.1007/s00281-018-0678-9 10.1007/s00281-018-0678-9 PMC6208671 29789863

[16] Sun L, Wang HX, Zhu XJ, Wu PH, Chen WQ, Zou P, et al Serum deprivation elevates the levels of microvesicles with different size distributions and selectively enriched proteins in human myeloma cells in vitro. Acta Pharmacol Sin [Internet]. Nature Publishing Group; 2014;35(3):381–3. Available from: 10.1038/aps.2013.166 24374813 PMC4647891

[17] Salomon C, Kobayashi M, Ashman K, Sobrevia L, Mitchell MD, Rice GE. Hypoxia-induced changes in the bioactivity of cytotrophoblast-derived exosomes. PLoS One. 2013;8(11). 10.1371/journal.pone.0079636 PMC3823597 24244532

[18] Nolte-’t Hoen ENM, van der Vlist EJ, de Boer-Brouwer M, Arkesteijn GJA, Stoorvogel W, Wauben MHM. Dynamics of dendritic cell-derived vesicles: high-resolution flow cytometric analysis of extracellular vesicle quantity and quality. J Leukoc Biol [Internet]. 2013;93(3):395–402. Available from: http://www.jleukbio.org/cgi/doi/10.1189/jlb.0911480 23248328 10.1189/jlb.0911480

[19] van der Vlist EJ, Arkesteijn GJA, van de Lest CHA, Stoorvogel W, Hoen ENMNt., Wauben MHM. CD4+ T cell activation promotes the differential release of distinct populations of nanosized vesicles. J Extracell Vesicles. 2012;1(1). 10.3402/jev.v1i0.18364 PMC3760647 24009884

[20] Kemball CC, Alirezaei M, Flynn CT, Wood MR, Harkins S, Kiosses WB, et al Coxsackievirus Infection Induces Autophagy-Like Vesicles and Megaphagosomes in Pancreatic Acinar Cells In Vivo. J Virol [Internet]. 2010;84(23):12110–24. Available from: http://jvi.asm.org/cgi/doi/10.1128/JVI.01417-10 20861268 10.1128/JVI.01417-10 PMC2976412

[21] Feng Z, Hirai-Yuki A, McKnight KL, Lemon SM. Naked Viruses That Aren’t Always Naked: Quasi-Enveloped Agents of Acute Hepatitis. Annu Rev Virol [Internet]. 2014;1(1):539–60. Available from: http://www.annualreviews.org/doi/10.1146/annurev-virology-031413-085359 26958733 10.1146/annurev-virology-031413-085359 PMC12175490

[22] McKnight KL, Xie L, González-López O, Rivera-Serrano EE, Chen X, Lemon SM. Protein composition of the hepatitis A virus quasi-envelope. Proc Natl Acad Sci [Internet]. National Academy of Sciences; 2017 6 20 [cited 2018 Jun 24];114(25):6587–92. Available from: http://www.pnas.org/content/114/25/6587 28490497 10.1073/pnas.1619519114 PMC5488923

[23] Hoen ENMNt., van der Vlist EJ, Aalberts M, Mertens HCH, Bosch BJ, Bartelink W, et al Quantitative and qualitative flow cytometric analysis of nanosized cell-derived membrane vesicles. Nanomedicine Nanotechnology, Biol Med [Internet]. Elsevier Inc.; 2012;8(5):712–20. Available from: 10.1016/j.nano.2011.09.006 PMC7106164 22024193

[24] van der Vlist EJ, Nolte-’t Hoen ENM, Stoorvogel W, Arkesteijn GJA, Wauben MHM. Fluorescent labeling of nano-sized vesicles released by cells and subsequent quantitative and qualitative analysis by high-resolution flow cytometry. Nat Protoc. 2012;7(7):1311–26. 10.1038/nprot.2012.065 22722367

[25] Carocci M, Bakkali-Kassimi L. The encephalomyocarditis virus. Virulence. 2012;3(4):351–67. 10.4161/viru.20573 22722247 PMC3478238

[26] Minciacchi VR, You S, Spinelli C, Morley S, Zandian M, Aspuria P, et al Large oncosomes contain distinct protein cargo and represent a separate functional class of tumor-derived extracellular vesicles. Oncotarget [Internet]. 2015;6(13):11327–41. Available from: http://www.ncbi.nlm.nih.gov/pubmed/25857301 25857301 10.18632/oncotarget.3598 PMC4484459

[27] Tkach M, Kowal J, Zucchetti AE, Enserink L, Jouve M, Lankar D, et al Qualitative differences in T-cell activation by dendritic cell-derived extracellular vesicle subtypes. EMBO J [Internet]. 2017 10 16;36(20):3012 LP–3028. Available from: http://emboj.embopress.org/content/36/20/3012.abstract 28923825 10.15252/embj.201696003 PMC5641679

[28] Tucher C, Bode K, Schiller P, Claßen L, Birr C, Souto-Carneiro MM, et al Extracellular vesicle subtypes released from activated or apoptotic T-lymphocytes carry a specific and stimulus-dependent protein cargo. Front Immunol. 2018;9(MAR):1–13. 29599781 10.3389/fimmu.2018.00534 PMC5862858

[29] Pols MS, Klumperman J. Trafficking and function of the tetraspanin CD63. Exp Cell Res [Internet]. Elsevier Inc.; 2009;315(9):1584–92. Available from: 10.1016/j.yexcr.2008.09.020 18930046

[30] Raposo G, Stoorvogel W. Extracellular vesicles: Exosomes, microvesicles, and friends. J Cell Biol. 2013;200(4):373–83. 10.1083/jcb.201211138 23420871 PMC3575529

[31] Andreu Z, Yáñez-Mó M. Tetraspanins in extracellular vesicle formation and function. Front Immunol. 2014;5(SEP):1–12. 25278937 10.3389/fimmu.2014.00442 PMC4165315

[32] Kowal J, Arras G, Colombo M, Jouve M, Morath JP, Primdal-Bengtson B, et al Proteomic comparison defines novel markers to characterize heterogeneous populations of extracellular vesicle subtypes. Proc Natl Acad Sci [Internet]. 2016;113(8):E968–77. Available from: http://www.pnas.org/lookup/doi/10.1073/pnas.1521230113 26858453 10.1073/pnas.1521230113 PMC4776515

[33] Osteikoetxea X, Sódar B, Németh A, Szabó-Taylor K, Pálóczi K, Vukman KV., et al Differential detergent sensitivity of extracellular vesicle subpopulations. Org Biomol Chem. Royal Society of Chemistry; 2015;13(38):9775–82. 10.1039/c5ob01451d 26264754

[34] Mitophagosomes E, Sin J, Mcintyre L, Stotland A, Feuer R, Gottlieb A. crossm Coxsackievirus B Escapes the Infected. 2017;91(24):1–16. 10.1128/JVI.01347-17 PMC5709598 28978702

[35] Yoshioka Y, Konishi Y, Kosaka N, Katsuda T, Kato T, Ochiya T. Comparative marker analysis of extracellular vesicles in different human cancer types. J Extracell Vesicles. 2013;2(1). 10.3402/jev.v2i0.20424 PMC3760642 24009892

[36] Belov GA, Van Kuppeveld FJ. (+)RNA viruses rewire cellular pathways to build replication organelles. Curr Opin Virol. Elsevier B.V.; 2012;2(6):734–41. 10.1016/j.coviro.2012.09.006 PMC7102821 23036609

[37] Strating JR, van Kuppeveld FJ. Viral rewiring of cellular lipid metabolism to create membranous replication compartments. Curr Opin Cell Biol. Elsevier Ltd; 2017;47:24–33. 10.1016/j.ceb.2017.02.005 PMC7127510 28242560

[38] Welsh JA, Holloway JA, Wilkinson JS, Englyst NA. Extracellular Vesicle Flow Cytometry Analysis and Standardization. Front Cell Dev Biol [Internet]. 2017;5(August):1–7. Available from: http://journal.frontiersin.org/article/10.3389/fcell.2017.00078/full 28913335 10.3389/fcell.2017.00078 PMC5582084

[39] Kormelink TG, Arkesteijn GJA, Nauwelaers FA, van den Engh G, Nolte-’t Hoen ENM, Wauben MHM. Prerequisites for the analysis and sorting of extracellular vesicle subpopulations by high-resolution flow cytometry. Cytom Part A. 2016;89(2):135–47. 10.1002/cyto.a.22644 25688721

[40] Wong J, Zhang J, Si X, Gao G, Mao I, McManus BM, et al Autophagosome Supports Coxsackievirus B3 Replication in Host Cells. J Virol [Internet]. 2008;82(18):9143–53. Available from: http://jvi.asm.org/cgi/doi/10.1128/JVI.00641-08 18596087 10.1128/JVI.00641-08 PMC2546883

[41] Jackson WT, Giddings TH, Taylor MP, Mulinyawe S, Rabinovitch M, Kopito RR, et al Subversion of cellular autophagosomal machinery by RNA viruses. PLoS Biol. 2005;3(5):0861–71. 10.1371/journal.pbio.0030156 PMC1084330 15884975

[42] Shu-Chen H, Chia-Lun C, Po-Shun W, Yu T, Hsiao-Sheng L. Enterovirus 71-induced autophagy detected in vitro and in vivo promotes viral replication. J Med Virol. 2009;81(7):1241–52. 10.1002/jmv.21502 19475621 PMC7166624

[43] Altan-Bonnet N. Extracellular vesicles are the Trojan horses of viral infection. Curr Opin Microbiol. Elsevier Ltd; 2016;32:77–81. 10.1016/j.mib.2016.05.004 PMC4983493 27232382

[44] Altan-Bonnet N, Chen Y-H. Intercellular Transmission of Viral Populations with Vesicles. J Virol. 2015;89(24):12242–4. 10.1128/JVI.01452-15 26423944 PMC4665251

[45] Zhu X, He Z, Yuan J, Wen W, Huang X, Hu Y, et al IFITM3-containing exosome as a novel mediator for anti-viral response in dengue virus infection. Cell Microbiol. 2015;17(1):105–18. 10.1111/cmi.12339 25131332 PMC7162390

[46] Li J, Liu K, Liu Y, Xu Y, Zhang F, Yang H, et al Exosomes mediate the cell-to-cell transmission of IFN-α-induced antiviral activity. Nat Immunol. 2013;14(8):793–803. 10.1038/ni.2647 23832071

[47] Khatua AK, Taylor HE, Hildreth JEK, Popik W. Exosomes Packaging APOBEC3G Confer Human Immunodeficiency Virus Resistance to Recipient Cells. J Virol [Internet]. 2009;83(2):512–21. Available from: http://jvi.asm.org/cgi/doi/10.1128/JVI.01658-08 18987139 10.1128/JVI.01658-08 PMC2612372

[48] Duke GM, Palmenberg AC. Cloning and synthesis of infectious cardiovirus RNAs containing short, discrete poly(C) tracts. J Virol. 1989;63(4):1822–6. 2538661 10.1128/jvi.63.4.1822-1826.1989 PMC248463

