# Supplementary figures and images for: Picornavirus infection induces temporal release of multiple extracellular vesicle subsets that differ in molecular composition and infectious potential
Source: PLoS Pathog. 2019 Feb 19;15(2):e1007594. doi: 10.1371/journal.ppat.1007594 (PMC6396942; doi:10.1371/journal.ppat.1007594)

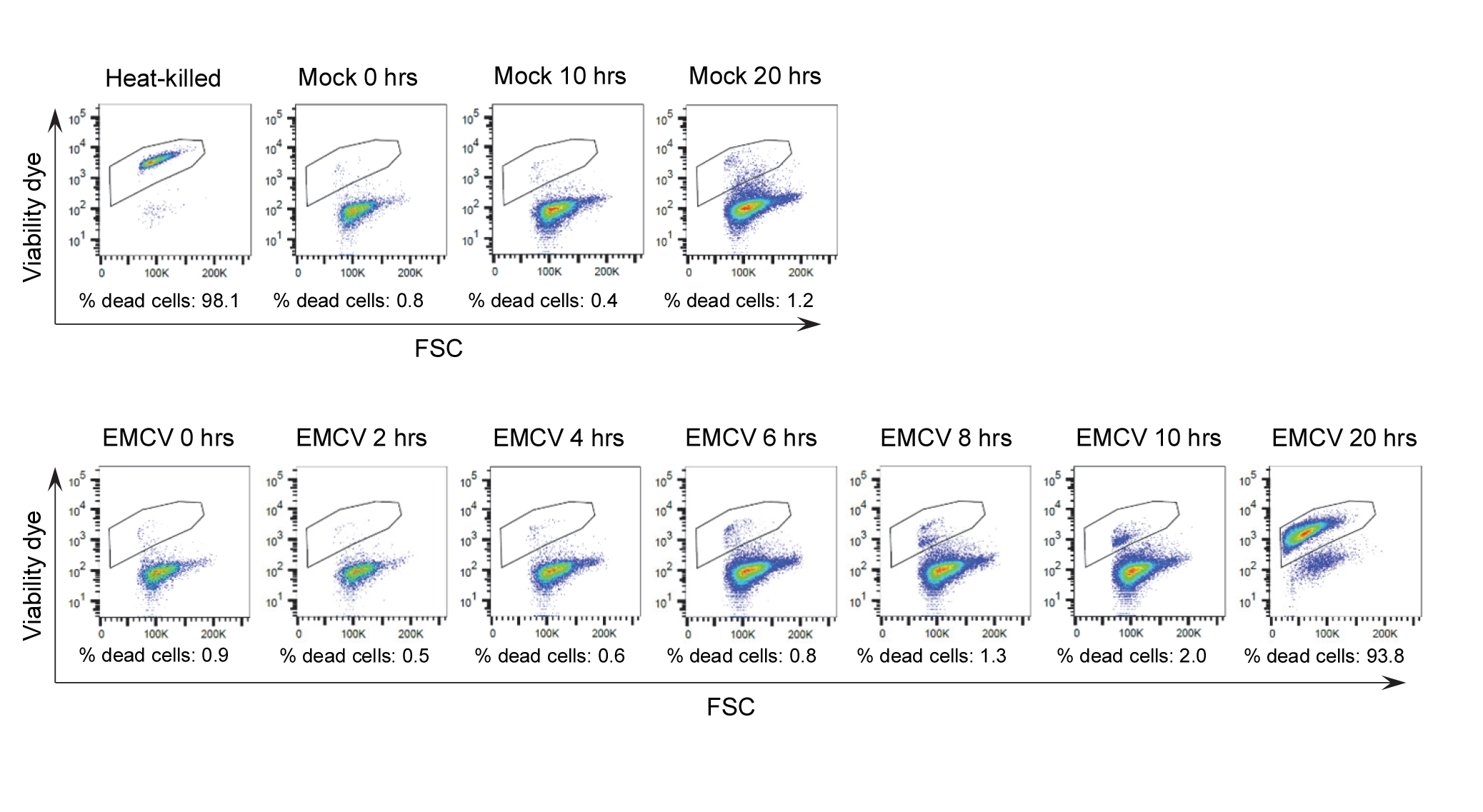

Supplement: S1 Fig — Flow cytometric analysis of mock cells and cells infected with EMCV stained with fixable Viability Dye eFluor780 after culture for the indicated amount of time. Heat killed cells were analyzed as a positive control, based on which a gate was set for eFluor780-positive events. For each condition, at least 20,000 events were recorded and the percentages of dead cells are indicated. Presented dot plots are representative of three experiments. (TIF) [file ppat.1007594.s001.tif]

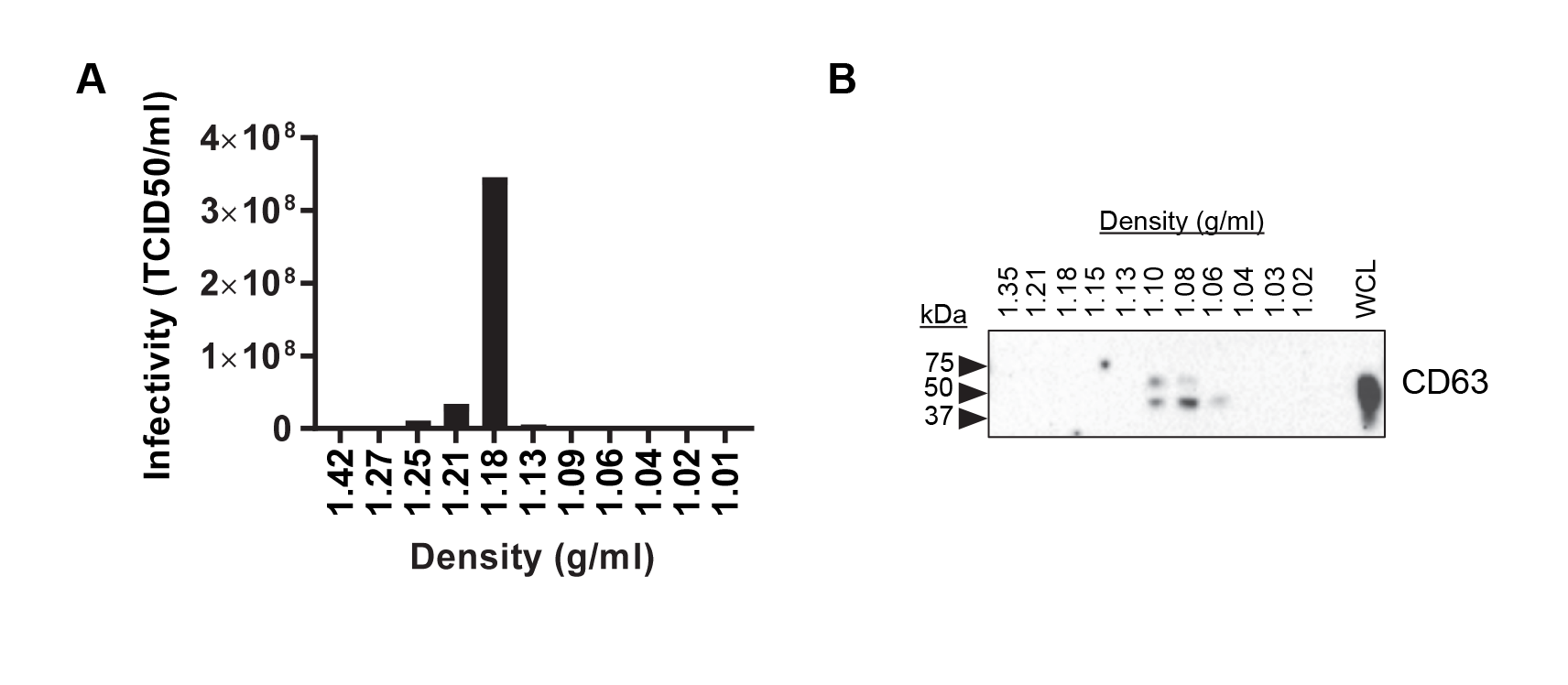

Supplement: S2 Fig — (A) EMCV virus particles harvested during the lytic phase of infection were treated with 0.1% triton to disrupt residual lipid membranes prior to buoyant density gradient centrifugation. Depicted is the infectivity in individual gradient fractions assessed by end-point dilution. (B) 100K EV from non-infected cells were separated on buoyant density gradients. Individual gradient fractions and control whole cell lysates (WCL) were analyzed for the presence of EV marker protein CD63 by western blotting. Presented are representative data of two independent experiments for A and B. (TIF) [file ppat.1007594.s002.tif]

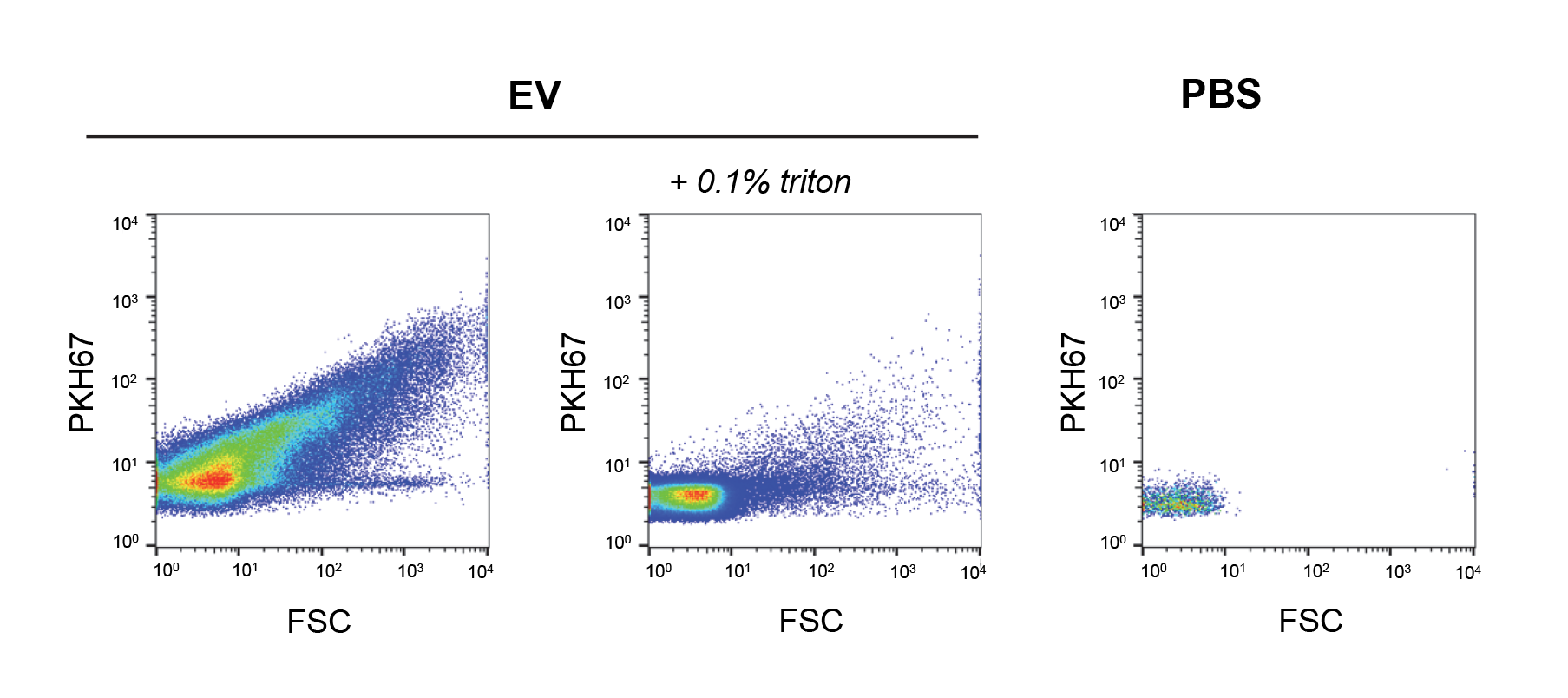

Supplement: S3 Fig — Efficiency of disruption of PKH67-labeled EV by treatment with 0.1% triton was assessed by high-resolution flow cytometry. Depicted are representative dot plots of control EV, triton-treated EV, or background events (PBS) detected above the fluorescence threshold during a 30 seconds acquisition. (TIF) [file ppat.1007594.s003.tif]

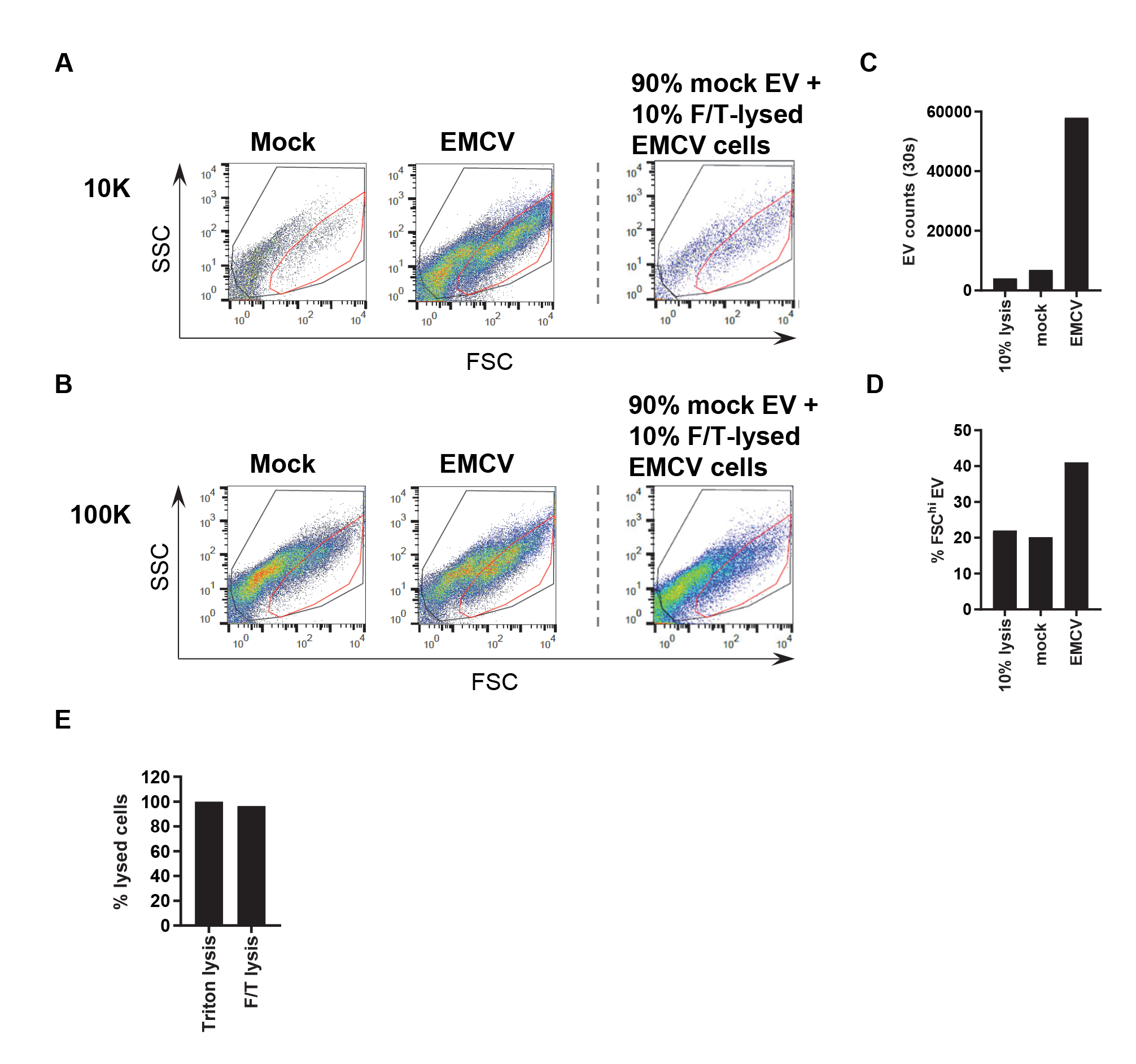

Supplement: S4 Fig — (A, B) 10K (A) and 100K (B) EV were isolated from supernatants of mock cells (left), EMCV-infected cells 8 hrs p.i. (middle), and mixed supernatants of lysed infected cells (10 v/v%) and mock cells (90 v/v%). EV were labeled with PKH67 and analyzed by high resolution flow cytometry. FSC-SSC plots represent quantitative flow cytometric measurements (30 seconds fixed time window) of EV in the 1.08 g/ml density fraction. (C, D) Bar graphs display the total number of 10K EV acquired during the 30 seconds measurements (C) and the percentage of FSChi EV of the total 100K EV detected in the indicated conditions (D). (E) Lysis of cells by freeze/thaw cycling was confirmed to be complete and comparable to triton-mediated lysis of cells by measuring leakage of the intracellular enzyme LDH into the extracellular space. Data are representative for two independent experiments. (TIF) [file ppat.1007594.s004.tif]

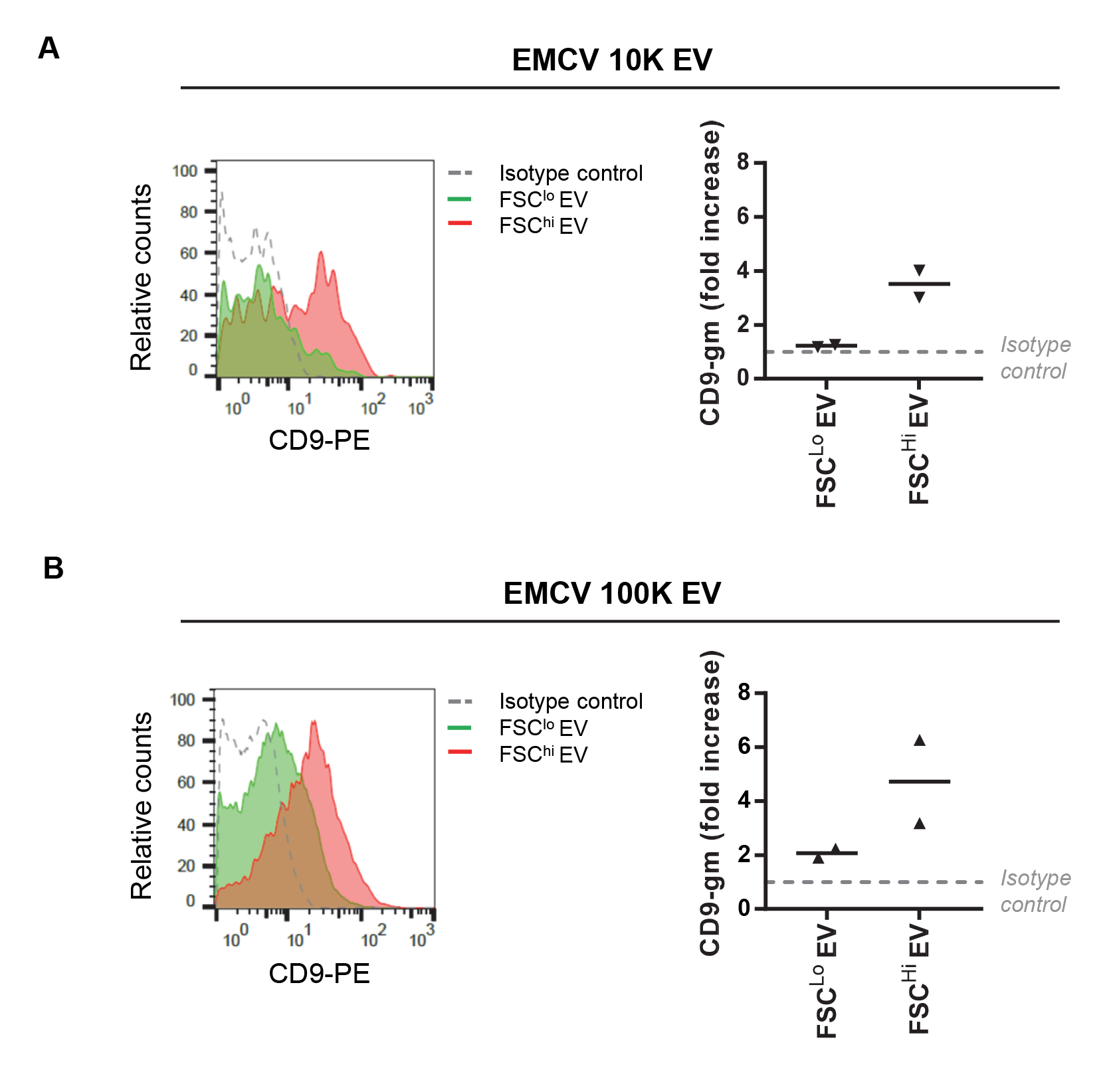

Supplement: S5 Fig — High resolution flow cytometric analysis of 10K (A) and 100K (B) EV concurrently labeled with PKH67 and PE-conjugated anti-CD9 or isotype control antibodies. Indicated are histogram overlays (left) and geometric mean fluorescence intensities (right) for CD9 relative to a matched isotype control detected on single FSChi or FSClo EV. (TIF) [file ppat.1007594.s005.tif]

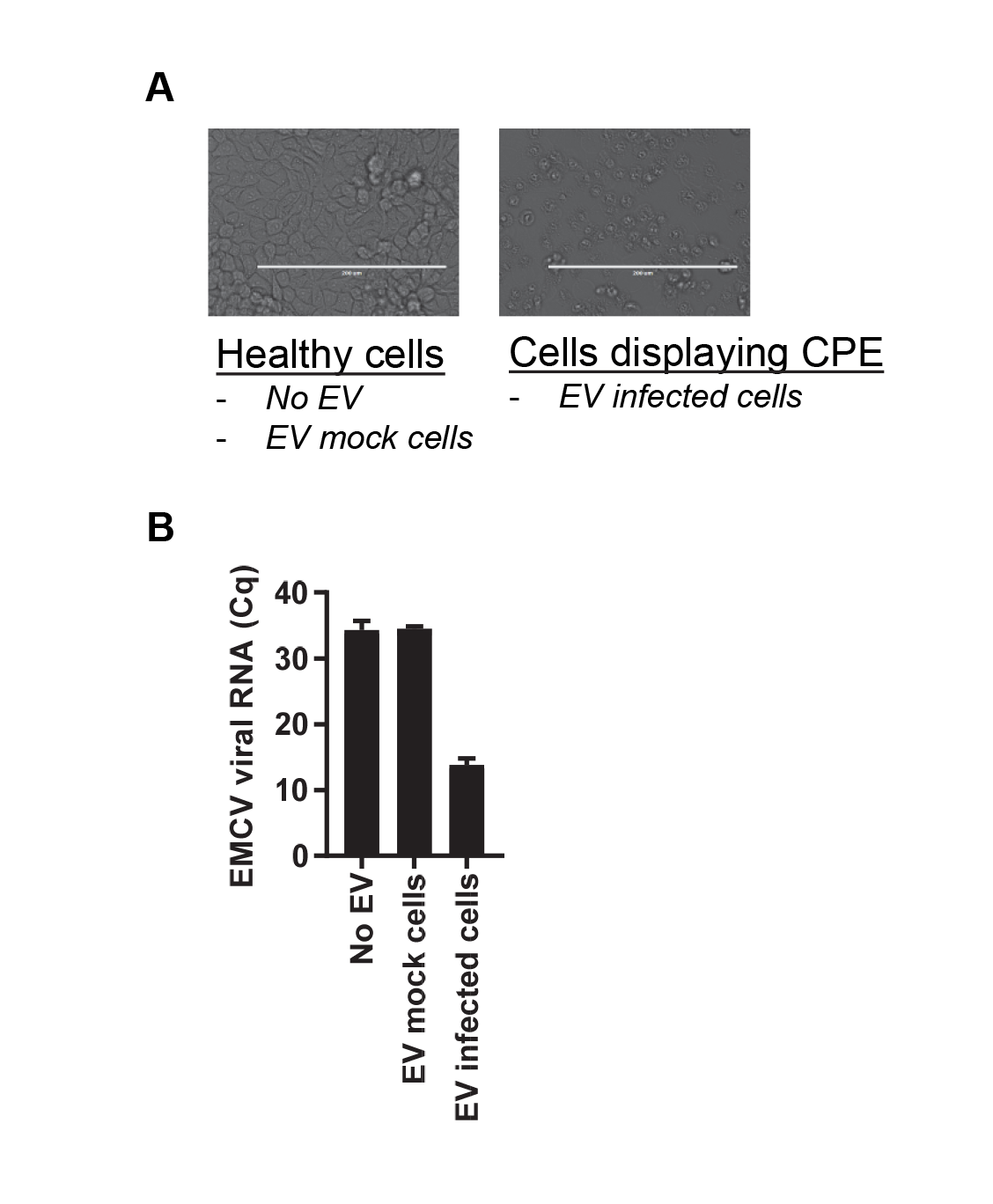

Supplement: S6 Fig — Viral genomic RNA levels in recipient cells of sort-purified EV subsets was assessed 3 days after sorting by RT-qPCR to confirm that the observed CPE was caused by EV-mediated transfer of infection and subsequent production of progeny virus. (A) Microscopic images showing recipient cells of EV that are healthy (left) or display CPE (right). Bar = 200 μm. (B) Cq values for viral genomic RNA in healthy cells that did not receive EV, healthy cells that received EV from mock-infected cells, and cells displaying CPE that received EV from EMCV-infected cells. Indicated are mean values ± s.d. for N = 3 independent experiments. (TIF) [file ppat.1007594.s006.tif]
